# The beta secretase BACE1 regulates the expression of insulin receptor in the liver

**DOI:** 10.1038/s41467-018-03755-2

**Published:** 2018-04-03

**Authors:** Paul J. Meakin, Anna Mezzapesa, Eva Benabou, Mary E. Haas, Bernadette Bonardo, Michel Grino, Jean-Michel Brunel, Christèle Desbois-Mouthon, Sudha B. Biddinger, Roland Govers, Michael L. J. Ashford, Franck Peiretti

**Affiliations:** 1 Division of Molecular & Clinical Medicine, Ninewells Hospital & Medical School, Dundee, DD19SY UK; 2 Aix Marseille Univ, INSERM, INRA, C2VN, 13385 Marseille, France; 3 Sorbonne Universités, UPMC Univ Paris 06, INSERM, Saint-Antoine Research Center, F-75012 Paris, France; 4 Division of Endocrinology, Boston Children’s Hospital, Boston, MA 02115 USA; 5 Aix Marseille Univ, INSERM, CNRS, CRCM, Institut Paoli Calmettes, Marseille, 13385 France

## Abstract

Insulin receptor (IR) plays a key role in the control of glucose homeostasis; however, the regulation of its cellular expression remains poorly understood. Here we show that the amount of biologically active IR is regulated by the cleavage of its ectodomain, by the β-site amyloid precursor protein cleaving enzyme 1 (BACE1), in a glucose concentration-dependent manner. In vivo studies demonstrate that BACE1 regulates the amount of IR and insulin signaling in the liver. During diabetes, BACE1-dependent cleavage of IR is increased and the amount of IR in the liver is reduced, whereas infusion of a BACE1 inhibitor partially restores liver IR. We suggest the potential use of BACE1 inhibitors to enhance insulin signaling during diabetes. Additionally, we show that plasma levels of cleaved IR reflect IR isoform A expression levels in liver tumors, which prompts us to propose that the measurement of circulating cleaved IR may assist hepatic cancer detection and management.

## Abstract (web summary)

A soluble form of insulin receptor in human plasma has been previously reported. Here the authors demonstrate that insulin receptor is cleaved by BACE1 that can regulate biological active insulin receptor levels in a glucose concentration-dependent manner, both in physiological and diabetic conditions.

## Introduction

Insulin receptor (IR) is a tetrameric protein composed of two extracellular ligand-binding α-subunits and two transmembrane tyrosine kinase active β-subunits^1^. IR exists as two isoforms, IRA and IRB, derived from the alternative splicing of exon 11 in the primary transcript^2^. IRA lacks and IRB contains a 12-amino acid segment located in the carboxyl terminus of the α-subunit. Both isoforms have similar affinity for insulin, but IRA also binds IGF2 with high affinity^3–6^. The relative abundance of the two variants is regulated in a tissue-specific manner^7^. IRA is ubiquitously expressed and is preponderant in fetal and cancer tissues as well as brain, whereas IRB is predominantly expressed in tissues associated with insulin-dependent metabolic effects (liver, muscle, and adipose tissue).

Mutations in *IR* gene, which reduce the number of cell-surface receptors, have been identified in patients with genetic syndromes of extreme insulin resistance (Donohue syndrome)^8^, suggesting that regulation of cell-surface IR levels contribute to the altered insulin signaling. Besides these rare genetic cases, IR cell-surface expression is also reduced in insulin-resistant states^9,10^, possibly consequential to its increased degradation^11,12^. IR overexpression with higher IRA levels are common features of most malignancies^7,13^ that may favor resistance to conventional and targeted therapies by various mechanisms^7^.

The presence of a soluble form of IR (full-length or truncated) in the conditioned media (CM) of several human cell lines and in human plasma was previously reported^14–18^. A landmark study^19^ demonstrated that a soluble truncated IR (IRsol), composed of the α-subunits attached to part of the extracellular region of β-subunits, was present at higher levels in the plasma of patients with diabetes than in control groups, a finding since been confirmed^20^. However, the molecular mechanisms responsible for IRsol generation remain unclear, and it is not known whether IR cleavage actively participates in the etiology of diabetes, and if diabetes is the only pathological situation in which IRsol is increased.

β-site amyloid precursor protein cleaving enzyme 1 (BACE1) is the transmembrane aspartyl protease required for the production of the neurotoxic β-amyloid peptide, considered crucial in the etiology of Alzheimer’s disease^21^. The propeptide of BACE1 is cleaved in the *trans*-Golgi network (TGN) by proprotein convertases, although immature BACE1 is active in the early biosynthetic compartments^22^. BACE1 is optimally active at acidic pH and is located in the TGN, plasma membrane, and early endosomes^23–25^. BACE1 has a loose substrate specificity^26^, and the use of published in vitro subsite specificity data, for a bioinformatics-based search of the human proteome, identified numerous putative BACE1 substrates^27^, including IR. Thus BACE1 may contribute to the regulation of insulin signaling. Importantly, BACE1 activity has been implicated in the regulation of whole-body glucose and energy homeostasis^28–30^.

We demonstrate that the IR ectodomain is cleaved by BACE1, that this cleavage occurs in the liver and increased during diabetes, thus decreasing the amount of mature IR. BACE1 inhibition restores functional cell-surface IR and increases insulin signaling, supporting the use of BACE1 inhibitors to improve liver insulin signaling during diabetes. Furthermore, IRsol plasma levels positively correlate with IRA expression in hepatic tumors, suggesting IRsol as a novel biomarker for cancer diagnosis, progression, and therapeutic efficacy.

## Results

### Identification of IR fragments

**Fig. 1:**
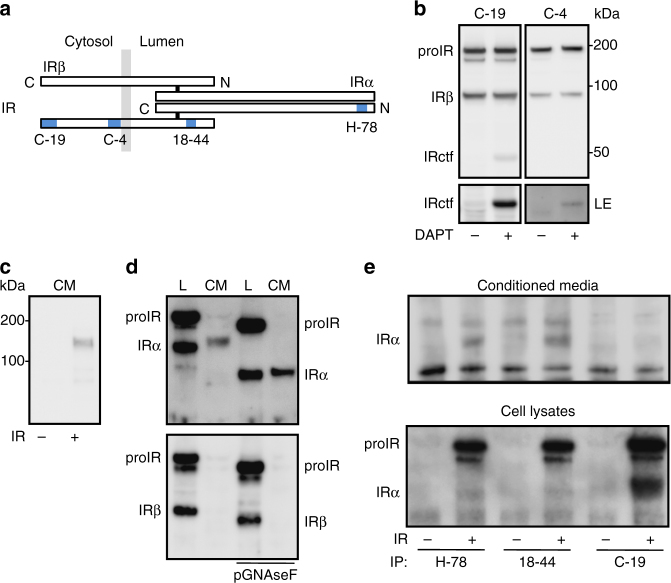
Detection and characterization of IR fragments. **a** Schematic representation of IR showing the position of the peptides used as antigens to produce antibodies C-19, C-4, H-78, and that of the epitope recognized by antibody 18-44 (blue rectangles). N and C-terminal extremities and α and β subunits are localized. The membrane is represented by the light gray rectangles. **b** HEK 293 cells were transfected with IR expression vector and treated overnight with the γ-secretase inhibitor (DAPT; 5 μM). IR was detected by immunoblot, using the indicated IR β-subunit (IRβ) specific antibodies (LE indicates a long exposure of the immunoblot). Positions of IR precursor (proIR), IRβ, and C-terminal fragment of IR (IRctf) are indicated. **c** Immunoblot analysis using the H-78 antibody of 24 h serum-free conditioned media (CM) from cells transfected with an empty plasmid (−) or with a plasmid encoding human IR (+). **d** Lysates (L) and CM from IR overexpressing cells were collected and where indicated in vitro, deglycosylated by PGNaseF. IR was detected by immunoblot, using H-78 antibody (upper panel) or C-19 antibody (lower panel). **e** CM and cell lysates from IR overexpressing cells (+) or cells transfected with an empty plasmid (−) were collected and subjected to immunoprecipitation (IP), using the indicated antibodies. IR was detected by immunoblot using H-78 antibody. IRα indicates the position of IR α-subunit

We validated various antibodies (Fig. 1a) to detect overexpressed wild type and truncated forms of IR in HEK 293 cells that express low levels of endogenous IR (Supplementary Fig. 1a, b). Throughout the overexpressed IR is isoform A (IRA; unless IRB specified).

The ectodomain cleavage of IR should generate a transmembrane C-terminal and a soluble fragment (IRctf and IRsol, respectively). IRctf is a substrate for presenilin-1-dependent activity of γ-secretase activity^31,32^, thus γ-secretase inhibition improves its detection. IRctf was detected with antibodies recognizing either the N or C-terminal intracellular domain of IR β-subunit (IRβ) (C-4 and C-19, respectively) at ≈50 kDa (Fig. 1b) in lysates of cells overexpressing IR and treated with the γ-secretase inhibitor (DAPT). The amount of IR precursor (proIR; an internal control of IR overexpression levels) and IRβ were not modified, indicating that HEK 293 cells express the enzyme that cleaves IR, and that IRctf contains the entire IR intracellular region. The transmembrane nature of IRctf was demonstrated by its detection in membrane-enriched fractions (Supplementary Fig. 2a) and in cell surface protein-enriched fractions (Supplementary Fig. 2b), implying that IRctf is generated by cleavage of the IRβ extracellular region, and that an ectodomain fragment of IR, containing α-subunit and a N-terminal β-subunit extracellular fragment is released into the extracellular media.

The IR α-subunit-specific antibody (H-78) detected a protein of ≈130 kDa in the CM of cells overexpressing IR that migrated slower in reducing SDS-PAGE than the cell lysate IR α-subunit (Fig. 1c, d, upper panel). The difference in migration distance persisted after the in vitro removal of N-linked oligosaccharides by Peptide-*N*-Glycosidase F (PNGaseF) treatment (Fig. 1d upper panel), suggesting it is not related to the *N*-glycosylation status. The detection of CM IR α-subunit was improved after deglycosylation, and this protein was not detected by the C-terminal IRβ-specific antibody (C-19) (Fig. 1d, lower panel). This protein was immunoprecipitated from CM by the IR α-subunit antibody (H-78) and the mouse monoclonal antibody 18-44^33^ that binds close to the β-subunit extracellular N-terminus (amino acids 792–797 from P06213), but not by the β-subunit intracellular C-terminal antibody (C-19) (Fig. 1e, upper panel). As expected, all antibodies immunoprecipitated IR from cell lysates (Fig. 1e, lower panel). Therefore, the protein detected by the IR α-subunit antibody (H-78) in CM is IRsol composed of the α-subunit and a β-subunit N-terminal fragment.

### BACE1 is responsible for cleavage of the IR ectodomain

We developed a bioluminescent reporter to monitor the cleavage of the extracellular region of IRβ consisting of Gaussia Luciferase fused to the N-terminus of IRβ (IRLuc), thus releasing Luciferase into the cell culture medium when cleaved (Supplementary Fig. 3a–c). IRLuc was used to screen for protease inhibitors that reduce the cleavage of the IRβ. Inhibitors of metalloproteases (GM6001) and ADAM17/MMP (TMI1) reduced Luciferase release (Supplementary Fig. 3d). BACE1 is not inhibited by pepstatin^34^, but treatment with its specific inhibitor (C3) reduced Luciferase release. None of these inhibitors modified the cellular amount of IRLuc (Supplementary Fig. 3d).

**Fig. 2:**
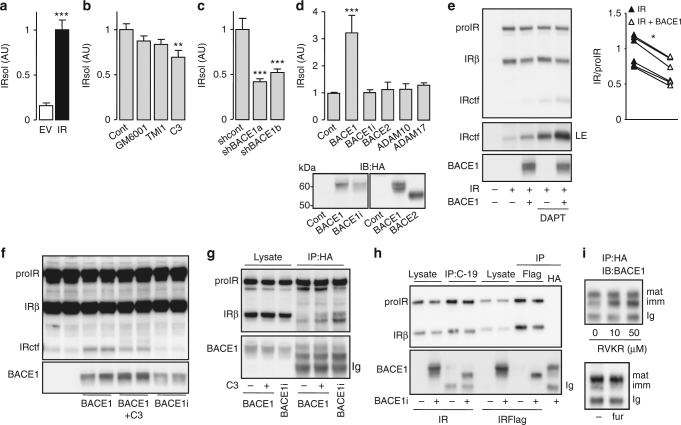
BACE1 is involved in IR cleavage. IRsol was measured by ELISA in 24 h conditioned media of **a** HEK 293 cells transfected with an empty vector (EV) or overexpressing IR. The mean IRsol values from IR overexpressing cells were arbitrarily set at 1. **b** Cells overexpressing IR and untreated (Cont) or treated with the indicated protease inhibitors (10 μM, 24 h). **c** Cells overexpressing IR together with BACE1 specific shRNA (shBACE1a, shBACE1b) or a control shRNA (shcont). **d** Cells transfected with the IR expression vector together with an empty plasmid (Cont), BACE1, inactive BACE1 (BACE1i), BACE2, ADAM10, or ADAM17. Expression of BACE1 and BACE2 were confirmed by immunoblot, using HA antibody (lower panel). **e** Cells transfected with combinations of IR and BACE1 expression vectors were treated or not with DAPT, then IR and BACE1 were detected by immunoblot. LE indicates a long exposure of the immunoblot. The graph shows the ratios IR/proIR obtained from the quantification of six independent experiments; data from the same experiment are connected by a line. **f** Cells overexpressing IR and BACE1 or inactive BACE1 (BACE1i) were treated with the BACE1 inhibitor (C3) then IR and BACE1 were detected by immunoblot. **g** Cells were treated as **f**, then BACE1 was immunoprecipitated (IP) with anti HA antibody and IR, and BACE1 were detected by immunoblot in cell lysates and immunoprecipitated fractions. **h** Cells were transfected with the indicated combinations of IR, IRFlag, and BACE1i expression vectors, then overexpressed proteins were immunoprecipitated using C-19, FlagM2 (Flag) and HA antibodies, respectively, and detected by immunoblots. **i** BACE1 overexpressing cells treated with proprotein convertase inhibitor dec-RVKR-cmk (RVKR; 24 h; upper panel) or cells overexpressing BACE1 without (−) of with furin (fur; lower panel) were lyzed then BACE1 was immunoprecipitated and detected by immunoblot. Positions of the heavy chain of the precipitating antibody (Ig), immature (imm) and mature (mat) BACE1 are indicated. Data are means ± s.d. Statistical analyses were made using unpaired *t*-test (**a**,** e**) or ANOVA followed by Dunnett’s test (**b**–**d**); **p < 0.01; ***p < 0.001

An ELISA, allowing measurement of human and mouse IRsol, was developed (Supplementary Fig. 4), and the three inhibitors that reduced IRLuc cleavage were studied. Overexpression of IR increased the amount of IRsol detected in CM (Fig. 2a) and IRsol accumulation was not altered by GM6001 or TMI1, but reduced by C3 (−30%) (Fig. 2b) and by two shRNAs specific for BACE1 (−50%) (Fig. 2c). The shRNAs efficiency to reduce BACE1 expression, and the lack of effect of the treatments on IR expression were verified (Supplementary Fig. 5a). The accumulation of IRsol in CM was increased by overexpression of BACE1, but not by an inactive form of BACE1 (BACE1i, a D_289_A mutant that is less well-expressed than the wild-type form), BACE2, ADAM10, or ADAM17 (Fig. 2d). The activity of overexpressed ADAM10 and ADAM17 was confirmed by increased cleavage of TNFα (Supplementary Fig. 5b)^35,36^. These results demonstrate that BACE1 activity selectively cleaves the IRβ ectodomain, releasing IRsol extracellularly.

The BACE1-dependent cleavage of IR was also analyzed in cell lysates. BACE1 overexpression reduced the amount of mature IR (Fig. 2e, left), decreasing the IR/proIR ratio (Fig. 2e, right), and increasing the amount of detectable IRctf even in the absence of DAPT (Fig. 2e, left). The increase in IRctf, by BACE1 overexpression, was reduced by C3 and the inactive form of BACE1 was unable to raise IRctf levels (Fig. 2f). Clearly, treatment of cells with C3 does not abolish BACE1 activity, in accordance with the modest effect of C3 on IRsol accumulation (Fig. 2b).

IR cleavage by BACE1 implies that the proteins interact. Immunoprecipitation (IP) of overexpressed BACE1 mainly pulled down the proIR and poorly its mature form (Fig. 2g). However, the amount of mature IR that co-immunoprecipitated with BACE1 increased when cells were incubated with C3 (Fig. 2g). Furthermore, more mature IR was co-immunoprecipitated with inactive BACE1 than wild-type BACE1, despite lower inactive BACE1 expression. These results favor a transient interaction between IR and BACE1 that is prolonged when BACE1 activity is reduced. BACE1 in cell lysates appeared as a poorly defined broad band in immunoblots, whereas two distinct bands were detected after BACE1 IP (Fig. 2g). Furthermore, IR IP only pulled down the fast migrating form of BACE1 (Fig. 2h), which corresponds to immature BACE1 (with its propeptide), as its amount was increased by protein convertases inhibition and decreased by furin overexpression (Fig. 2i). Consequently, as previously shown^23^, the slow migrating form of BACE1 is mature BACE1 (without its propeptide).

The interaction of IR with immature BACE1 suggests that IR cleavage occurs in the early secretory pathway. This notion is supported by the fact that BACE1, IR, and IRctf co-purify in Golgi/TGN-enriched fractions, and that IRsol is detected in cell lysates (Supplementary Fig. 6).

### BACE1 regulates cell surface IR amount and insulin signaling

**Fig. 3:**
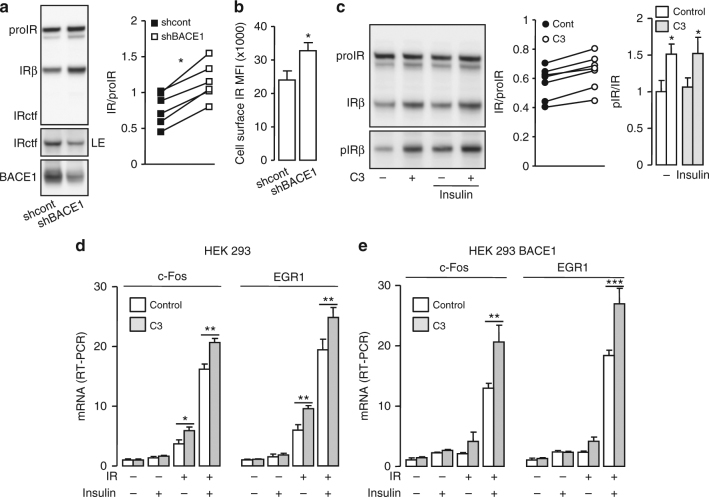
BACE1 regulates the amount of cell surface IR. **a** HEK 293 cells, stably expressing BACE1, were co-transfected with IR expression vector and control shRNA (shcont) or BACE1 specific shRNA (shBACE1) then IR and BACE1 were analyzed by immunoblot. LE indicates a long exposure of the immunoblot. The graph shows the ratio IR/proIR obtained from the quantification of six independent experiments; data from the same experiment are connected by a line. **b** IR expression was measured by flow cytometry at the surface of cells, stably expressing BACE1 and co-transfected with the IR expression vector associated with control shRNA (shcont) or BACE1 specific shRNA (shBACE1). The median fluorescence intensity (MFI) corrected for the value obtained with cells transfected with the empty vector is shown. **c** Cells expressing BACE1 were transfected with IR expression vector and treated overnight with the BACE1 inhibitor (C3; 20 μM) in the absence of serum, then stimulated with insulin (5 nM, 5 min) and IR, and phospho IR (pIRβ) were analyzed by immunoblot. The graphs show the ratios IR/proIR and pIR/IR obtained from the quantification of seven and six independent experiments, respectively. Native HEK 293 cells (**d**) or HEK 293 cells expressing BACE1 (**e**) were transfected with IR coding vector (+) or with empty vector (−), treated with BACE1 inhibitor (C3; 20 μM; gray columns), serum deprived for 20 h and stimulated with insulin (5 nM, 45 min; +). Levels of c-Fos and Egr1 mRNA were measured by RT-PCR and expressed as fold over the situation without IR overexpression nor insulin stimulation. Data are means ± s.d. Statistical analyses were made using unpaired *t*-test (**a**–**c**) and ANOVA followed by Bonferroni’s test (**e**); *p < 0.05; **p < 0.01

In BACE1 overexpressing cells, BACE1 knockdown (Fig. 3a) reduced the amount of IRctf, but increased mature IR levels (increased IR/proIR ratio; +54%) and cell surface IR (+40%) (Fig. 3b). Similarly, treatment of cells with C3 (Fig. 3c) reproducibly increased mature IR levels (increased IR/proIR ratio; +14 ± 4%). Furthermore, basal and insulin-stimulated IR phosphorylation (Tyr_1162/1163_) increased proportionally to the amount of IR with pIR/IR ratios unaltered by BACE1 inhibition. Thus BACE1-dependent IR cleavage modulates the amount of biologically active IR at the cell surface. Importantly, BACE1-dependent IR cleavage was confirmed in human hepatoma HepG2 cells, which express substantial endogenous IR (Supplementary Fig. 7), showing that it is not restricted to cells ectopically overexpressing IR and/or BACE1.

In HEK 293 cells, insulin-induced expression of immediate-early genes (*c-Fos* and *EGR-1*) was proportional to the amount of overexpressed kinase-competent IR and involved the Erk1/2 signaling pathway (Supplementary Fig. 8). Inhibition of BACE1 increased insulin-stimulated *c-Fos* and *EGR-1* expression only in cells expressing IR and was enhanced in cells overexpressing BACE1 (Fig. 3d, e). These results illustrate that the increase in IR amount triggered by BACE1 inhibition enhances insulin action.

### Identification of IR cleavage region

**Fig. 4:**
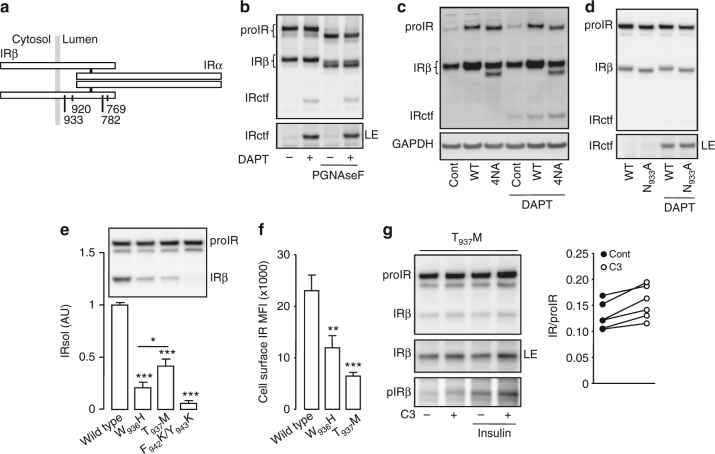
Identification of the IR cleavage region. **a** Schematic representation of IR positioning the *N*-glycosylation sites in the β-subunit. **b** HEK 293 cells were transfected and treated as described in Fig. 1b. Where indicated cell lysates were in vitro deglycosylated by PGNaseF. **c** CHO cells were transfected with an empty plasmid (Cont), an IR expression vector (WT) or with a plasmid expressing a mutated form of IR that cannot be glycosylated on its β-subunit (4NA), then treated with DAPT. **d** HEK 293 cells were transfected with an IR expression vector (WT) or with a plasmid expressing a mutated form of IR that cannot be glycosylated on the position 933 (N_933_A), then treated with DAPT. HEK 293 cells expressing BACE1 were transfected with wild-type IR or the indicated mutated forms of IR, then the IRsol accumulated in the conditioned media was measured by ELISA, and cellular expression of IR was analyzed by immunoblot (**e**), cell surface expression of IR was measured by flow cytometry (**f**). **g** Cells were treated as in Fig. 3c then IR and phospho IR (pIRβ) were detected by immunoblot. LE indicates a long exposure of the immunoblot. The graph shows the ratios IR/proIR obtained from the quantification of six independent experiments. Data are means ± s.d. Statistical analyses were made using ANOVA, followed by Bonferonni’s test (**e**, **f**) or unpaired *t*-test (**g**). *p < 0.05; **p < 0.01; ***p < 0.001

The IRβ contains four *N*-glycosylation sites (Fig. 4a)^37^. In vitro enzymatic *N*-deglycosylation of cell lysates increased the migration distance of the IRβ, but not IRctf, in SDS-PAGE (Fig. 4b), showing that the IRctf extracellular region is not *N*-glycosylated. For confirmation, CHO cells were transfected with vectors coding for wild-type IR or modified IR, in which the four *N*-glycosylatable asparagine residues in the IRβ were replaced by alanine. CHO cells express substantial levels of endogenous mature IR; however overexpressed IR was readily detectable (Fig. 4c). Abrogation of the IRβ glycosylation increased its migration distance in SDS-PAGE with IRctf unchanged (Fig. 4c). Asparagine in position 933 is the most membrane proximal *N*-glycosylation site in IRβ, and mutation to alanine modestly increased the migration distance of IRβ but not IRctf (Fig. 4d). These results indicate that IRβ cleavage occurs in its membrane proximal stalk, between asparagine 933 and lysine 956 (Supplementary Table 1). The calculated molecular weight of the IRβ C-terminal fragment generated by cleavage in this region lies between 48.1 and 50.7 kDa, which is compatible with the mass of IRctf deduced from SDS-PAGE migration (49–51 kDa).

A BACE1 target motif was predicted^27^ within the IR region we have identified (Supplementary Table 1). A peptide encompassing part of this motif was in vitro cleaved by recombinant soluble BACE1, generating fragments with RP-HPLC elution profiles compatible with cleavage at the predicted scissible bond (Supplementary Fig. 9), indicating direct cleavage of IR by BACE1.

Amino acid substitutions were performed (Supplementary Table 1) to identify the crucial residues for cleavage. The scissible bond amino acids were substituted (FY to KK) to generate the IR mutant F_942_K/Y_943_K expected to be resistant to BACE1 cleavage^38^. The preferred amino acid for BACE1-dependent cleavage, in the P_7_ position (tryptophan), was substituted by the unfavorable amino acid (histidine)^39^ to generate the IR mutant W_936_H with reduced cleavage efficiency expected. The P_6_ threonine was changed to methionine to generate the IR mutant T_937_M, reported in two cases of Donohue syndrome^40,41^.

The amount of IRsol generated from each mutant was lower than that from wild type (Fig. 4e). However, these mutants were mainly expressed as precursors (Fig. 4e, inset), suggesting an impairment in their cellular trafficking. Mutant F_942_K/Y_943_K was almost exclusively expressed as a precursor and generated minute amounts of IRsol, which underscores a major alteration of its cellular trafficking and therefore was not studied further. Compared with the IR mutant W_936_H, T_937_M generated more IRsol (Fig. 4e), its mature form was less abundant (Fig. 4e, inset), and it was less well-expressed at the cell surface (Fig. 4f). These results suggest that cleavage of T_937_M is more efficient than that of W_936_H. As for wild-type IR (Fig. 3c), BACE1 inhibition reproducibly increased mature IR T_937_M levels (IR/proIR; +17 ± 7%) and that of its phosphorylated form in response to insulin stimulation (Fig. 4g).

### Regulation of BACE1-dependent IR cleavage by glucose

**Fig. 5:**
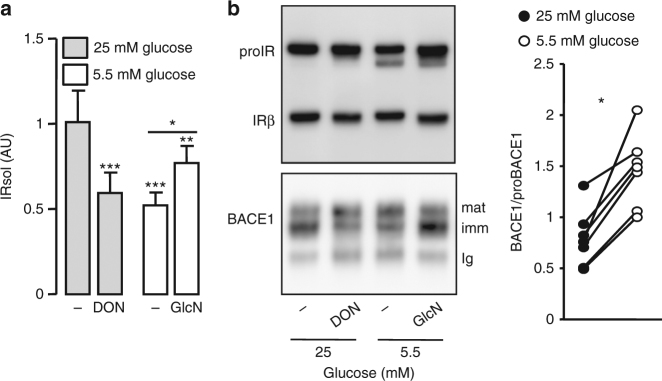
Effect of glucose on IR cleavage and BACE1 expression. HEK 293 cells expressing IR and BACE1 were incubated for 24 h in media containing the indicated glucose concentrations, in the absence (−) or presence of deoxynorleucine (DON; 5 mM) or glucosamine (GlcN; 1 mM) then: **a** IRsol accumulated in the media was measured by ELISA; **b** IR and BACE1 were analyzed by immunoblot in total cell lysate or after BACE1 immunoprecipitation, respectively. Positions of the heavy chain of the precipitating antibody (Ig), immature (imm), and mature (mat) BACE1 are indicated. The graph shows the ratios BACE1/proBACE1 obtained from the quantification of seven different experiments; data from the same experiment are connected by a line. Data are means ± s.d. Statistical analyses were made using ANOVA, followed by Bonferonni’s test (**a**) and unpaired *t*-test (**b**). *p < 0.05; ***p < 0.001

Reducing the glucose concentration of HEK 293 cells culture media from 25 to 5.5 mM reduced IRsol accumulation (Fig. 5a) and the cleavage of the IR cleavage reporter system (decreased accumulation of Luciferase in culture media and increased cellular Luciferase) (Supplementary Fig. 10a). Addition of glucosamine (GlcN) (a hexose substrate specifically metabolized through the hexosamine biosynthesis pathway and *O*-GlcNAcylation candidate) mitigated the low glucose-dependent reduction of IRsol accumulation (Fig. 5a). Conversely, in a high glucose concentration, global inhibition of *O*-GlcNacylation by deoxynorleucine (glucose:fructose amidotransferase inhibitor) reduced IRsol accumulation (Fig. 5a). These treatments modified overall cellular *O*-GlcNacylation (Supplementary Fig. 10b), but not the amount of mature IR (Fig. 5b, upper panel), suggesting that IR cleavage regulation by glucose involves *O*-GlcNacylation processes.

Low glucose concentration reduced the expression level of immature BACE1 (the form that interacts with IR) and increased that of mature BACE1, resulting in an increased BACE1/proBACE1 ratio (Fig. 5b). Similar effects were triggered by inhibition of *O*-GlcNacylation, while addition of GlcN abolished the effects of low glucose (Fig. 5b and Supplementary Fig. 10c). However, proprotein convertase activity was not altered by low glucose (Supplementary Fig. 10d). These results suggest that immature BACE1 is responsible for IR cleavage and that glucose regulates the amount of BACE1 that is matured by proprotein convertases.

### In vivo cleavage of IR by BACE1

**Fig. 6:**
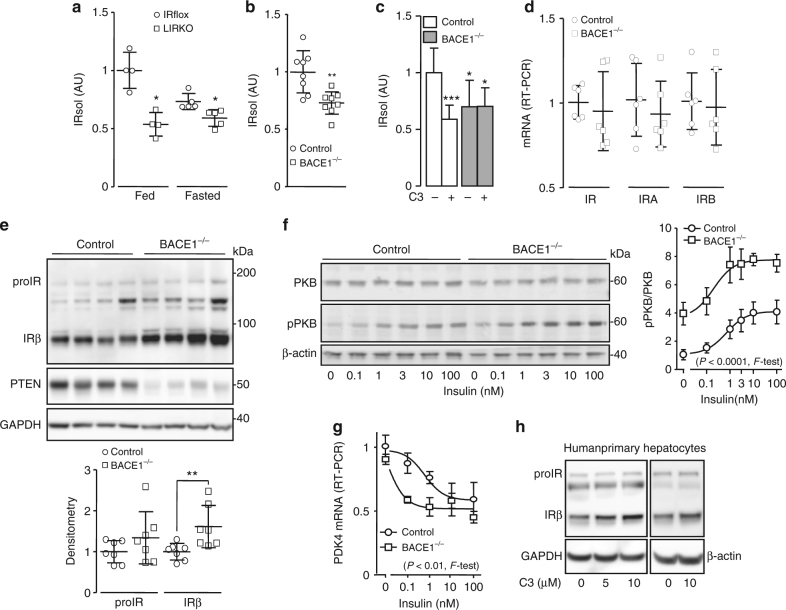
In vivo cleavage of IR by BACE1. IRsol was measured in the plasma from **a** liver-specific IR knockout mice (LIRKO; fed *n* = 4; fasted *n* = 5)and their floxed control (IRflox; fed *n* = 4; fasted n = 5), **b** BACE1^−/−^ mice (*n* = 8) and their control littermates (*n* = 8). **c** IRsol was measured in the conditioned media from control and BACE1^−/−^ liver explants treated or not with the BACE1 inhibitor (C3). **d** Levels of IR, IRA, and IRB mRNA were measured by RT-PCR in control and BACE1^−/−^ livers. **e** Levels of IR, PTEN, and GAPDH (loading control) were analyzed by immunoblot in control and BACE1^−/−^ livers, a densitometric analysis of the proIR and IRβ bands normalized to GAPDH was performed (below the blot), values are expressed as fold over the means of the wild-type mice. **f** Primary hepatocytes isolated form control (*n* = 4) and BACE1^−/−^ (*n* = 3) mice were stimulated for 10 min with the indicated concentration of insulin. A representative immunoblot of insulin-stimulated phosphorylation of PKB at Ser^473^ is shown. The curves on the right of the immunoblot are the normalized means ± s.e.m of the immunoblots (both curves differed significantly, *p* < 0.0001, *F*-test). **g** mRNA levels of PDK4 were measured by RT-PCR in control and BACE1^−/−^ primary hepatocytes stimulated for 6 h with the indicated concentrations of insulin (both curves differed significantly, *p* < 0.01, *F*-test). **h** Human primary hepatocytes (two different preparations) were incubated for 40 h in the presence of the indicated concentrations of BACE1 inhibitor (C3), then IR and GAPDH or actin (taken as loading controls) were analyzed by immunoblot. Data are means ± s.d. Statistical analyses was made using Mann–Withney (**a**), unpaired *t*-test (**b**,** d**, **e**), ANOVA followed by Dunnett’s test (**c**) or *F*-test (**f**,** g**) *p < 0.05; **p < 0.01; ***p < 0.001

Significantly less IRsol was measured in the plasma of liver-specific IR knockout mice than their floxed-controls (Fig. 6a), suggesting that a substantial amount of plasma IRsol is produced by the liver. Interestingly, fasting reduced IRsol plasma levels only in wild-type mice (Fig. 6a). Plasma from BACE1^−/−^ mice contained less IRsol (−30%) than the plasma from wild-type littermates (Fig. 6b), confirming the involvement of BACE1 in the cleavage of IR in vivo. Furthermore, liver explants from BACE1^−/−^ mice released less IRsol than liver explants from wild-type mice (Fig. 6c), and C3 reduced the IRsol release only in wild-type liver explants, corroborating hepatic BACE1-dependent IR cleavage. Total IR, IRA, and IRB mRNA levels were similar in livers from wild-type and BACE1^−/−^ mice (Fig. 6d). However, BACE1^−/−^ mouse livers contained more (+60%) mature IR (Fig. 6e and Supplementary Fig. 11a). Protein levels of the PKB negative regulator PTEN are reduced by IR overexpression^42^, and in accordance we detected low PTEN levels in BACE1^−/−^ livers (Fig. 6e and Supplementary Fig. 11a). In primary hepatocytes, BACE1 deficiency increased total IR and phosphorylated IR levels (basal and insulin stimulated) (Supplementary Fig. 12a) without altering mRNA (Supplementary Fig. 12b). In contrast to the other BACE1 reduction models used in this study, the proform of IR was increased in BACE1^−/−^ primary hepatocytes (Supplementary Fig. 12a). Basal and insulin-stimulated phosphorylation of PKB were increased in BACE1^−/−^ hepatocytes (Supplementary Fig. 12a), with insulin concentration–response curve for PKB phosphorylation shifted upward, with an increased maximal response (Fig. 6f). Furthermore, repression of *PDK4* occurred with lower concentrations of insulin in BACE1^−/−^ than control hepatocytes (leftward shift in insulin concentration–response curve) (Fig. 6g), while the insulin repression of *PEPCK*, *G6Pase,* and *PGC1a* expression were unchanged (Supplementary Fig. 12c). These results show that BACE1 deficiency in primary hepatocytes improves some insulin effects. Importantly, BACE1-dependent cleavage of IR occurs in primary human hepatocytes, as the treatment with C3 increased the amount of mature IR (Fig. 6h).

**Fig. 7:**
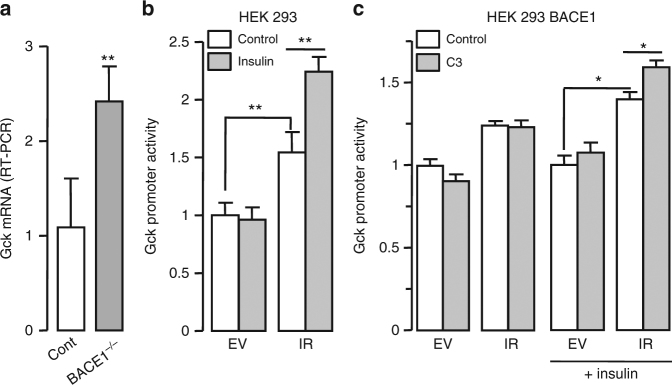
Regulation of glucokinase expression by IR amount. **a** Levels of GCK mRNA were measured by RT-PCR in control and BACE1^−/−^ primary hepatocytes. Native HEK 293 cells (**b**) or HEK 293 cells overexpressing BACE1 (**c**) were transfected with empty vector (EV) or with IR coding vector together with human GCK promoter-driven Firefly luciferase and SV40-driven Renilla luciferase coding vectors where indicated, serum-deprived cells were treated with the BACE1 inhibitor (C3; 20 μM) and then stimulated with insulin (2 nM, 7 h). Firefly and Renilla luciferase were measured in cell lysates, and GCK promoter activity was calculated as the ratio of Firefly/Renilla luciferase and expressed as fold over the situation without IR overexpression nor insulin stimulation. Data are means ± s.d. Statistical analyses were made using unpaired *t*-test (**a**) or ANOVA, followed by Bonferonni’s test (**b**, **c**). *p < 0.05; **p < 0.01

A restricted analysis of expression of genes, involved in glucose and lipid metabolism (Supplementary Fig. 12b), revealed that basal glucokinase (*GCK*) mRNA was higher in BACE1^−/−^ than control primary hepatocytes (Fig. 7a). A luciferase reporter gene expression system was used to evaluate whether *GCK* promoter activity was depended on IR expression levels. Human GCK promoter activity was increased in IR overexpressing cells and insulin-stimulated promoter activity only in these cells (Fig. 7b). In BACE1 overexpressing cells, both IR overexpression and insulin stimulation were required to increase GCK promoter activity, which was further enhanced by BACE1 inhibition (Fig. 7c). This result shows that BACE1-dependent cleavage of IR is involved in the regulation of GCK expression.

### Cleavage of IR in diabetic mice

**Fig. 8:**
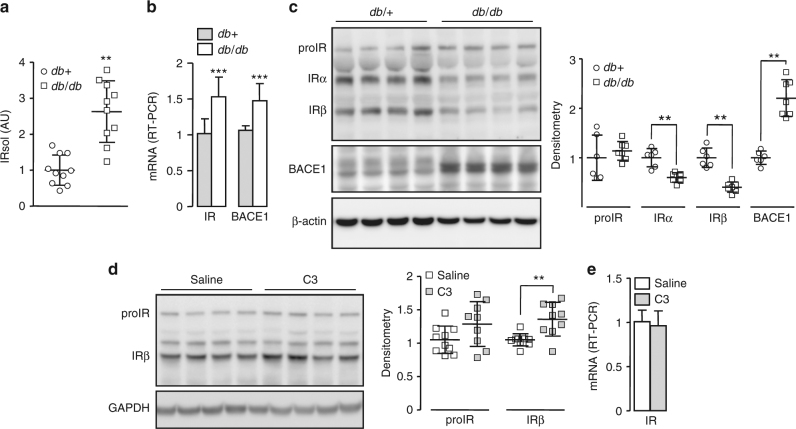
IR cleavage in a mouse model of diabetes. **a** IRsol was measured in the plasma from *db/db* mice (*n* = 10) and from their control littermate (*db/+*; *n* = 10). **b** mRNA levels of IR and BACE1 were measured by RT-PCR in the liver. **c** Liver expression of IR, BACE1, and β-actin (loading control) were analyzed by immunoblot; a densitometric analysis of the proIR, IRα, IRβ, and BACE1 bands was performed (right), values are expressed as fold over the means of the wild-type mice. **d** *db/db* mice were treated with saline or with BACE1 inhibitor (C3, 4 weeks), then liver expression of IR and GAPDH (loading control) were analyzed by immunoblot; a densitometric analysis of the proIR and IRβ bands was performed (right). **e** mRNA levels of IR were measured by RT-PCR in the liver of *db/db* mice treated with saline or with the BACE1 inhibitor (C3). Data are means ± s.d. Statistical analyses were made using unpaired *t*-test. **p < 0.01; ***p < 0.001

We detected more IRsol in the plasma of a model of type 2 diabetes (*db/db* mice, Supplementary Fig. 13a) than in plasma from the control (*db/+*) (Fig. 8a). As the liver contributes to BACE1-dependent IRsol generation, BACE1 and IR expression were analyzed in this organ. BACE1 mRNA and protein levels were increased in *db/db* mouse livers (Fig. 8b, c and Supplementary Fig. 11b), suggesting increased BACE1 activity. Interestingly, IR mRNA levels were increased (Fig. 8b), whereas the amount of mature IR was reduced (Fig. 8c and Supplementary Fig. 11b), implying that transcription-independent mechanisms are involved in the regulation of mature IR expression. Similar regulation of plasma IRsol levels, liver IR and BACE1 mRNA levels, and protein amounts was observed in high-fat diet (HFD) fed mice with impaired glucose tolerance, compared with normal-fat diet fed mice (Supplementary Fig. 13a–d). For comparison, IR and BACE1 mRNA levels were not increased in the epididymal fat of *db/db* and HFD fed mice (Supplementary Fig. 13e).

Treatment of *db/db* mice with C3 increased the amount of hepatic mature IR (+35%) (Fig. 8d and Supplementary Fig. 11c) without altering IR mRNA levels (Fig. 8e). These results support the notion that BACE1-dependent cleavage of IR contributes to the reduced liver IR expression in *db/db* mice. However, C3 treatment had no impact on plasma IRsol levels (control = 1 ± 0.29 AU (arbitrary units); BACE1 inhibitor = 0.88 ± 0.42 AU) or on blood glucose (control = 18.2 ± 1.2 mmol/L; BACE1 inhibitor = 17.8 ± 1.3 mmol/L).

### Cleavage of IR in tumors

**Fig. 9:**
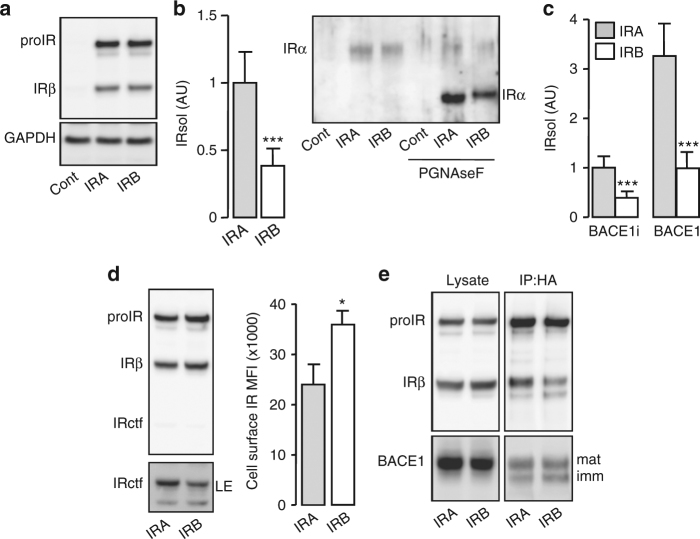
BACE1-dependent cleavage of IRA and IRB. **a** HEK 293 cells were transfected with an empty vector (Cont) or with IRA or IRB expression vector, then IR and endogenous GAPDH expression were detected by immunoblot. **b** IRsol was measured in conditioned media of cell expressing IRA or IRB by ELISA (left) and by immunobloting of the conditioned media untreated or treated with PGNaseF. **c** Cells were transfected with the indicated combinations of IRA, IRB and BACE1, and inactive BACE1 (BACE1i) expression vectors; then IRsol was measured by ELISA in conditioned media. **d** BACE1 overexpressing cells were transfected with IRA or IRB expression vector and IR was detected by immunoblot (left) or at the cell surface by flow cytometry (right). LE indicates a long exposure of the immunoblot. **e** Inactive BACE1 was co-immunoprecipitated with IRA or IRB as described in Fig. 2g. Immature (imm) and mature (mat) BACE1 are indicated. Data are means ± s.d. Statistical analyses were made using unpaired *t*-test. *p < 0.05; ***p < 0.001

We compared cleavage efficiencies of the two IR isoforms. Similar amounts of IRA and IRB were overexpressed in HEK 293 cells (Fig. 9a), but the amount of IRsol in CM of cells overexpressing IRA was approximately twice that in CM of cells overexpressing IRB (Fig. 9b). Treatment of CM with PGNaseF improved the detection of IRsol by immunoblot and showed that IRB migrates slower than IRA, which was expected since the α-subunit of IRB is 12-amino acids longer than that of IRA.

Overexpression of BACE1 increased shedding of IRA and IRB by three- and twofold, respectively (Fig. 9c). In BACE1 overexpressing cells, the amounts of mature IRA and IRB were similar; however, more IRctf was detected in IRA than IRB overexpressing cells, and IRB was more highly expressed at the cell surface than IRA (Fig. 9d). Furthermore, mature IRB was less efficiently immunoprecipitated with inactive BACE1 than IRA (Fig. 9e). These results show that BACE1-dependent cleavage of IRA is more efficient than that of IRB.

**Fig. 10:**
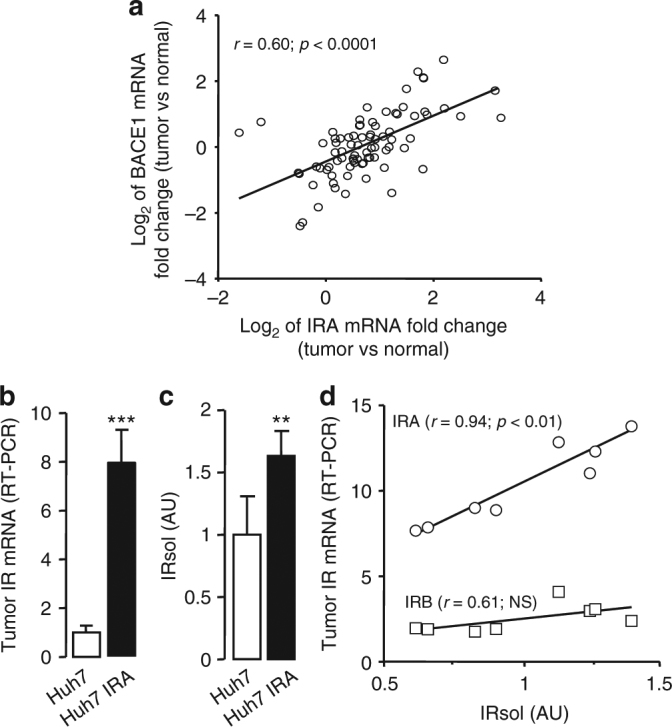
IR cleavage in tumors. **a** Pearson’s correlation of the changes in IRA mRNA levels in hepatocellular carcinoma vs. normal tissue with those of BACE1 (values are expressed in Log_2_). Pearson’s correlation coefficient (*r*) and *p*-value are reported. **b** IR mRNA levels were measured by RT-PCR in tumors from native Huh7 cells and from Huh7 cells overexpressing IRA (Huh7 IRA). **c** IRsol was measured in the plasma of mice-bearing tumors from native Huh7 cells and from Huh7 cells overexpressing IRA (Huh7 IRA). Data are means ± s.d. **p < 0.01; ***p < 0.001. **d** Correlation of IRA (circles) and IRB (squares) mRNA levels in tumors from native Huh7 cells with IRsol plasma levels (IRsol values are expressed as fold over the mean). Pearson’s correlation coefficient (*r*) and *p*-value are reported (NS, no significant correlation)

As overexpression of IR, with higher levels of IRA, are frequently observed in malignant tumors, we determined whether facilitated cleavage of IRA was observed in this context. We first examined expression levels of IR and BACE1 in 85 hepatocellular carcinomas and paired the surrounding non-tumor liver tissues. The majority of tumors (80%) had increased IRA mRNA, and changes in tumor IRA expression were positively correlated with BACE1 expression, suggesting that these tumors efficiently cleave IRA and produce IRsol (Fig. 10a).

To highlight a potential relationship between tumor IRA expression and IRsol generation, we xenotransplanted the human hepatocarcinoma cell line Huh7 (native and overexpressing IRA) in nude mice; and after tumor development, tumor IR expression and plasma IRsol levels were measured. Plasma from mice-bearing IRA-overexpressing tumors (Fig. 10b) contained more IRsol than plasma from mice-bearing tumors from native cells (Fig. 10c), while there was no difference in mice body weight (23.3 ± 2.9 g vs 22.2 ± 1.6 g), tumor volumes (1.23 ± 0.43 cm^3^ vs 1.26 ± 0.16 cm^3^), and BACE1 mRNA levels (1.0 ± 0.16 vs 1.05 ± 0.31). In tumors originating from native Huh7 cells, IRA mRNA levels accounted for 76 ± 6% of the total IR mRNA and correlated with IRsol plasma levels (Fig. 10d). No association was observed between IRB mRNA and IRsol plasma levels. These results indicate that IRA expressed in a tumor is primarily responsible for the production of IRsol detectable in plasma.

## Discussion

Our results demonstrate that the protease BACE1 cleaves the ectodomain of IR, thereby regulating its cell surface expression. This regulation occurs in vivo, with a significant contribution from the liver and is particularly altered during diabetes.

We confirmed, as previously proposed, that IR undergoes proteolysis that generates a soluble fragment (IRsol) composed of its α-subunits attached to portions of its extracellular β-subunits^20^ and a transmembrane fragment of its β-subunits (IRctf), the latter being fully degraded by the successive activities of γ-secretase and proteasome^19,32^. A screening of protease inhibitors suggested that BACE1 is involved in IR cleavage. The selective cleavage of IR by BACE1 was further confirmed by showing that knockdown and overexpression of BACE1 reduced and increased IR cleavage, respectively, whereas overexpression of BACE2 (a close homolog of BACE1), ADAM10 or ADAM17 did not increase IR cleavage. The involvement of BACE1 in IR cleavage is further supported by the detection of a transient interaction between the two proteins that necessarily precedes IR cleavage. This interaction involves immature BACE1, which possesses enzymatic activity^22^. BACE1, IR and IRctf co-purify in Golgi/TGN-enriched fractions. In addition IRsol is detectable in cell lysates. Taken together, these findings suggest that BACE1-dependent IR cleavage occurs in the lumen of the Golgi/TGN, thereby rationalizing detection of the interaction between the proIR and immature BACE1. We hypothesize that these BACE1 and IR forms interact in the early secretory pathway before the action of proprotein convertase in the TGN^22,43^. Interestingly, IRctf cleavage by γ-secretase also occurs in the TGN and, when γ-secretase is inhibited, a fraction of the IRctf reaches the cell surface. We show that reducing the glucose concentration decreases IR cleavage, which is congruent with previous data showing that BACE1 activity^44^ and IR shedding^45^ are stimulated by high glucose levels. As previously described^45^, we show that regulation of IR cleavage by glucose involves *O*-GlcNacylation. Furthermore, reducing glucose concentration decreases the amount of immature BACE1, while increasing the mature form in an *O*-GlcNacylation-dependent manner. Our interpretation is that immature BACE1 cleaves IR and that high glucose triggers the *O*-GlcNacylation processes that reduce the amount of BACE1 matured by proprotein convertases (without interfering with proprotein convertase activity), thereby increasing IR cleavage (Supplementary Fig. 14). Identification of the *O*-GlcNacylation processes responsible for this regulation is beyond the scope of the current work.

BACE1 interacts more with IRA than IRB, with IRA more readily cleaved than IRB. The exon 11-encoded 12-amino acid peptide in IRB may be responsible for the decreased interaction between IRB and BACE1. Alternatively, the favored interaction between BACE1 and IRA may be due to their coincident presence in particular sub-membrane domains. Indeed, BACE1 and IR were reported to be in lipid rafts^46–48^ and localization of IR isoforms in separate lipid raft microdomains has been documented^49,50^.

The BACE1-dependent increased IR cleavage is associated with decreased amounts of its mature form. Conversely, BACE1 reduction increases IR cell surface expression, resulting in an insulin-dependent increased IR phosphorylation and enhanced insulin signaling effectiveness as denoted by higher expression of the insulin-stimulated immediate-early genes *c-Fos* and *EGR-1*. These results are consistent with recent data that emphasize the importance of the amount of cell surface IR in the control of IR signaling^11,51,52^.

We have demonstrated that IR is cleaved in the extracellular region of its β-subunits (between amino acids 933 and 956), a region containing a predicted BACE1 target motif^27^, and that a peptide containing part of this sequence is cleaved in vitro by recombinant soluble BACE1. However, amino acid substitutions in this region of IR disrupt its intracellular trafficking, which prevents the exact molecular identification of the BACE1 cleavage sequence but reveals the importance of the integrity of this region in IR biology. Interestingly, despite its reduced intracellular trafficking the IR mutant T_937_M, reported in two cases of Donohue syndrome^40,41^, is efficiently cleaved by BACE1. Consequently, inhibition of BACE1 increases the amount of its mature form, which is phosphorylated upon insulin stimulation.

The low levels of plasma IRsol of BACE1^−/−^ and of liver-specific IR knockout mice validate the role of BACE1 in the cleavage of IR in vivo and indicate that the liver contributes significantly to IRsol plasma levels. Furthermore, IRsol release from liver explants is reduced by deficiency or inhibition of BACE1, confirming BACE1-dependent IR cleavage in the liver and suggesting that this cleavage can be drug targeted. As expected, more mature IR is detected in the liver of BACE1^−/−^ mice than in control mice. In addition, expression of the PKB negative regulator PTEN is reduced in BACE1^−/−^ livers and since high levels of IR decrease PTEN expression^42^, it is conceivable that the increased expression of IR in BACE1^−/−^ livers is partly responsible for the low PTEN levels. Consequently, we suggest that the increased insulin-dependent phosphorylation of PKB in the liver of BACE1^−/−^ mice, that we have previously described^28^, involves both increased IR levels and derepression of PKB phosphorylation. A higher level of IR is also observed in primary BACE1^−/−^ hepatocytes with evidence of some enhanced insulin signaling processes (raised PKB phosphorylation, repression of PDK4 expression). BACE1^−/−^ hepatocytes also display increased levels of *GCK* mRNA and we show that *GCK* promoter activity depends on regulation of IR by BACE1 activity. Thus BACE1-dependent regulation of liver IR levels may alter insulin effectiveness. As we used primary hepatocytes that show normal insulin sensitivity, some of the insulin effects may already be optimal (repression of *PEPCK*, *G6pase*, and *PGC1α*) and not improved by BACE1 deficiency. It is likely that diminished PTEN levels underlying the derepression of PKB phosphorylation contribute in increased basal levels of IR and PKB phosphorylation in BACE1^−/−^ hepatocytes. However, despite the high basal PKB phosphorylation, insulin-stimulated phosphorylation amplitude is conserved, raising the total level of phosphorylated PKB. Importantly, we show that BACE1-dependent IR cleavage also occurs in human primary hepatocytes.

Mouse models of diabetes (*db/db*) and impaired glucose tolerance (HFD fed mice) have increased plasma levels of IRsol, consistent with observations made in patients with diabetes^19,20^. BACE1 expression is increased in the livers of these mice, in agreement with increased liver BACE1 activity in mice with impaired glucose tolerance^28^, and the amount of mature IR is post-transcriptionally reduced. Treatment of *db/db* mice with C3 increases hepatic levels of the mature form of IR, while remaining below (approximately half) of those of the control mice, indicating that BACE1 activity is involved in the reduction of liver IR expression that occurs during diabetes. Pharmacological inhibition of BACE1 is relatively ineffective in *db/db* mice as denoted by no decrease in IRsol plasma levels, and lack of improvement in glucose homeostasis, suggesting a role for leptin signaling^53^. However, whole-body deletion or inhibition of BACE1 improves the insulin sensitivity of HFD fed mice^28,29^. A neuron-specific human BACE1 knockin mouse model provides evidence that increased neuronal BACE1 expression is sufficient to cause systemic diabetic complications^54^. Therefore, peripheral metabolic disturbances caused by increased BACE1 levels and activity may be, at least in part, secondary to central impairment. Nevertheless, during diabetes the BACE1-dependent cleavage of liver IR, by reducing hepatic insulin sensitivity, may also contribute to peripheral metabolic disorder. Taken together, our results support the repurposing of BACE1 inhibitors, currently in clinical trials for Alzheimer’s disease, to recover some active IR in the liver of patients with diabetes, thus reducing hepatic insulin resistance.

A short period of fasting reduces IRsol plasma levels in wild type but not in liver-specific IR knockout mice, suggesting that IRsol plasma levels are regulated by the nutritional status and this regulation predominantly involves BACE1-dependent cleavage of liver IR. The hexosamine biosynthetic pathway responsible for protein *O*-GlcNacylation plays a central role in sensing the nutritional status of the cell^55^ and high global *O*-GlcNacylation levels were reported in the liver of *db/db* and HFD fed mice^56^. Therefore, as shown in our cellular model herein, we propose that in mice, *O*-GlcNacylation processes are the basis of regulation of IR cleavage in the liver.

We and others have reported that tumors from different tissue origin have increased expression of IRA^7,13^, which may favor resistance to conventional and targeted therapies by a variety of mechanisms^7^. We show that in human hepatocellular carcinomas, changes in IRA expression are positively correlated with those of BACE1, implying that these tumors are competent to cleave IR. In addition, xenograft experiments show that IRsol plasma levels mainly depend on the expression levels of IRA in the tumor. Taken together, our results may explain why IRsol plasma levels are increased in patients with cancers (published in patent WO 2004/097414) and suggest IRsol as a useful biomarker for tumor IRA expression levels that could help diagnose and monitor cancer evolution and even guide therapy.

In summary, our data demonstrate for the first time that IR is a novel substrate for BACE1 and that therapies targeting BACE1 inhibition could be an innovative treatment for diabetes and other diseases characterized by insulin resistance. Moreover, IRsol may also be considered a cancer biomarker and assist rational decisions making in cancer disease management.

## Methods

### Reagents

Inhibitors of BACE1 (C3), presenilin (DAPT) and proprotein convertases (dec-RVKR-cmk) were purchased from Merck (Nottingham, UK). TMI1 was synthesized (Supplementary Methods). 6-diazo-5 oxo-l-Norleucine and GlcN were from Sigma Aldrich. All other inhibitors, IR antibodies C-19, C-4, H-78 and phosphospecific (Tyr_1162/11636_), PTEN (A2B1), HA-probe (Y-11) and *O*-GlcNac (CTD110.6) antibodies were from Santa Cruz Biotechnology (Santa Cruz, CA, USA). IR antibodies 18-44 and 83-7 (biotin labeled) were from Thermo Fisher Scientific. Antibodies specific for Golgin 97 (CDF4), PKB (C67E7), pPKB (193H12), Erk1/2 (9102), pErk1/2 (9101), Presenilin-1 (D39D1), Nicastrin (D38F9), β-actin (13E5), and GAPDH (D4C6R) were from Cell Signaling Technology (Danvers, MA, USA). Anti-GFP (mixture of clones 7.1 and 13.1) was from Roche. Anti-BACE1 (B0681) and Anti-FlagM2 were from Sigma Aldrich.

### Expression vectors

The expression vectors for IRA, IRB, non-glycosylatable form of IRA, GFP-tagged IRβ, HA-tagged BACE1 and BACE2, ADAM17, ADAM10, TNF-α and the vectors containing human BACE1 shRNA were described elsewhere^35,37,49,57–59^. The intracellular fragment of IR was generated by PCR amplification and cloned into pcDNA3 expression vector. Mutated forms of IR (N_933_A, W_936_H, T_937_M, F_942_K/Y_943_K and A_946-949_), inactive form of BACE1 (D_289_A)^47^ and mutated form of BACE1 (T_47_A) were created using the GeneArt site-directed mutagenesis system (Thermo Fisher). Expression vector for IRβ N-terminally fused to Gaussia Luciferase was generated by homologous recombination using the In-fusion cloning system (Clontech).

### Cell culture and transfection

HEK 293 cells (Griptite 293 MSR) were from Thermo Fisher Scientific. CHO, HepG2 and Huh7 cell lines were from ATCC. The cells were maintained in culture as described by the manufacturers. No mycoplasma contamination was detected in any of the cultures. Transient and stable cell transfection were performed with PolyJet reagent (SignaGen Laboratories, Rockville, MD, USA) and Lipofectamine 3000 (Thermo Fisher), respectively, as specified by the manufacturers.

### Mouse studies

Liver-specific insulin receptor knockout mice (LIRKO; Alb-Cre^+/−^, IR lox/lox) mice and their littermate flox controls (IRflox; Cre^−/−^, IR lox/lox) were maintained on a mixed genetic background as previously described^60,61^, and housed with a 07:00–19:00 lights-on light cycle. Blood was collected at 14:00 from 2–3-month-old male mice in either the non-fasted state or after a 5 h fast and plasma was prepared. Animal experiments were performed with the approval of the Institutional Animal Care and Research Advisory Committee at Children’s Hospital Boston (USA).

BACE1^−/−^ mice were previously described^28^. Blood was collected from overnight fasted mice by cardiac puncture then plasma was prepared. Liver was removed and pieces were snap frozen for ulterior analysis of IR expression level. The rest of the liver was minced into 2 mm per piece. Fifteen to twenty pieces of liver explants were incubated in DMEM with or without C3 (1 μM) for 24 h in a humidified incubator at 37 °C. Medium was replaced and incubated for a further 24 h. Liver explants were collected, drained, and weighed; CM was collected and analyzed for IRsol content. Primary hepatocytes were isolated from adult mice using a collagenase method^62^. The cells were plated in M199 medium supplemented with 100 μg/mL penicillin–streptomycin, 0.1% bovine serum albumin, 10% FBS, 200 nM dexamethasone, 100 nM triiodothyronine, 10 nM insulin, at a density of 2.5 × 10^5^ cells/well in six-well plates. After attachment (3–4 h), hepatocytes were maintained in M199 medium with antibiotics and 100 nM dexamethasone for 16 h before use.

Experiments were performed at the University of Dundee (UK) in accordance to the Animal Scientific Procedures Act (1986), with approval of Universities of Dundee Ethic Committee.

Male *db/db* (*BKS.Cg-m+/+Leprdb/J*) and *db/+*(*BKS.Cg-m+/−Leprdb/J*) mice (Charles River Laboratories; Saint Germain Nuelles, France) were 10 weeks old at the time of killing. For in vivo BACE1 inhibition experiment, 8 weeks old *db/db* mice were randomly implanted with osmotic minipumps (Alzet; model 2004) containing C3 (10 mg/kg/day) or vehicle (50:50 DMSO/PBS) then killed 28 days later. Ten weeks old male C57Bl/6 mice (Charles River Laboratories; Saint Germain Nuelles, France) were randomly fed either a standard-fat diet (70% kcal carbohydrate, 10% kcal fat, 20% kcal protein, 3.68 kcal g^−1^, Special Diet Services, Witham, UK) or a HFD (20% kcal carbohydrate, 60% kcal fat, 20% kcal protein, 5.13 kcal g^−1^) for 16 weeks before killing. Blood and tissues were collected as described above. Experiments were performed at Aix Marseille University (France) in accordance to the European directive 2010/63/EU on the protection of animals used for scientific purposes and approved by the “Comité d'éthique en expérimentation animale de Marseille.”

Six-weeks old female (Hsd: athymic Nude-Foxn1nu) mice (Envigo) were inoculated *s.c*. in the flank with 2 × 10^6^ Huh7 cells suspended in 50% Matrigel (BD Biosciences, San Jose, CA). After tumor development, mice were anesthetized by isoflurane inhalation, blood was collected by cardiac puncture and plasma was prepared. Tumors were removed, weighted, and snap frozen for ulterior analysis of IR expression level. Xenograft experiments were performed at Paris-Sorbonne University (France) in accordance to the European directive 2010/63/EU on the protection of animals used for scientific purposes and approved by the “Direction départementale des services vétérinaires de Paris.”

### Human liver tissue specimens

Eighty-five hepatocellular carcinoma and paired adjacent non-tumor liver tissues were collected with informed consent from patients undergoing curative liver resection at the Saint-Antoine Hospital and stored in a tumor biobank (Pathology department, Saint-Antoine hospital) in accordance to the French laws and regulations (Commission Nationale de l’Informatique et des Libertés no. 1913901 v0). Clinicopathologic characteristics of the 85 patients have been published previously^63^.

### Enzymatic deglycosylation

N-linked carbohydrate residues were removed by incubating cell lysates or serum-free CM for 2 h at 37 °C with 1000 units of PNGaseF as described by the manufacturer (New England Biolabs, Beverly, MA, USA).

### ELISA

TNFα was measured in CM as described by the manufacturers (R&D Systems).

For the detection of IRsol, plasma or conditioned culture media were centrifuged to remove cells and cell debris then filtered through 0.2 μM nylon membrane (Ceveron MFU 500 filter plate; Technoclone) to remove microparticles. ELISA plate (SpectraPlate-HB; Perkin Elmer) was coated with the monoclonal antibody 18-44^64^ specific for the extracellular portion of the IRβ (2 μg/mL in Coating Buffer pH 7.4; Candor-Bioscience) overnight at 4 °C. The plate was washed twice with dilution buffer (Super Block Blocking buffer from Thermo Fisher Scientific + Tween 20 0.05%) then incubated 0.5 h at room temperature in dilution buffer. Diluted samples were incubated in wells overnight at 4 °C. After several washes, the biotinylated monoclonal antibody 83-7^64^ specific for the extracellular IR α-subunit (1/750 in dilution buffer) was incubated for 2 h at room temperature. After several washes, HRP-labeled streptavidin in dilution buffer was added and incubated 0.5 h. After several washes, a tyramide signal amplification protocol (Elast ELISA Perkin Elmer) was applied as described by the manufacturer. HRP substrate (TMB Super Sensitive One Component from SurModics) was added to the wells and OD was monitored at 650 nm until the high inter assay control reach a value of 0.5, then the reaction was stopped by adding equal volume 0.6 N sulfuric acid and OD was measured at 450 nm. Changes in OD 450 nm values were expressed relative to the mean of reference values, which was set to 1. IRsol in the figures is thus expressed as AU.

### Immunoblot

Identical volumes of microparticle-free CM (serum free) and plasma or identical amounts of total protein were heat-denatured and reduced (70 °C; 10 min) then submitted to SDS-PAGE separation on 4–12% gradient NuPAGE gels (Life Technologies, Saint Aubin, France) and transferred to polyvinylidene fluoride membranes. Membranes were blocked for 1 h in 5% BSA solution and incubated with the appropriate primary and HRP-conjugated secondary antibodies (1:1000 and 1:10,000 dilution, respectively). Immunodetections were performed using ECL reagent and image acquisition was performed by using a chemiluminescent CCD imager ImageQuant LAS 4000 (GE Healthcare, Velizy-Villacoublay, France). Densitometric analysis of the bands was performed with the ImageQuant TL software (GE Healthcare, Velizy-Villacoublay, France).

### Flow cytometry

Cell surface expression of overexpressed IR was analyzed by flow cytometry (Accury C5) using the biotinylated anti IRα-subunit antibody (83-7) and streptavidin Alexafluor 488 (Thermo Fisher Scientific). Cells were gated on forward and side scatter to exclude dead cells, debris and aggregates.

### Real-time PCR analysis

Total RNA was extracted using Nucleospin RNA Kit (Macherey-Nagel, Hoerdt, France), cDNA was synthesized from 0.5 µg of RNA using M-MLV reverse transcriptase (Life Technologies, Saint Aubin, France) and used for PCR amplification. RT-PCR were performed on the LightCycler 480 instrument (Roche Applied Science, Meylan, France) using the Eva Green MasterMix (Euromedex, Souffelweyersheim, France). The comparative Ct method (2^−(ΔΔCT)^) was used to calculate the relative differences in mRNA expression. The acidic ribosomal phosphoprotein P0 or the hypoxanthine-guanine phosphoribosyltransferase was used as housekeeping gene. Primers sequences are available upon request. Changes were normalized to the mean of control values, which were set to 1.

### Statistical analyses

All data were analyzed with GraphPad Prism software and individual statistical two-sided tests used are identified in the figure legends. *P*-values ≤ 0.05 were considered statistically significant. Mice experiments were exploratory; there was no estimation to base the effective sample size.

### Data availability

The data supporting the findings of this study are available within the article and Supplementary File, or available from the corresponding author on reasonable request.

## Electronic supplementary material

Supplementary Information(PDF 1080 kb)
