# Supplementary material for: The beta secretase BACE1 regulates the expression of insulin receptor in the liver
Source: Nat Commun. 2018 Apr 3;9:1306. doi: 10.1038/s41467-018-03755-2 (PMC5880807; doi:10.1038/s41467-018-03755-2)
Supplement: Supplementary file 1 — Supplementary Information(PDF 1080 kb) [file 41467_2018_3755_MOESM1_ESM.pdf]

Supplementary Information

The beta secretase BACE1 regulates the expression of insulin receptor in the liver

Meakin et al.

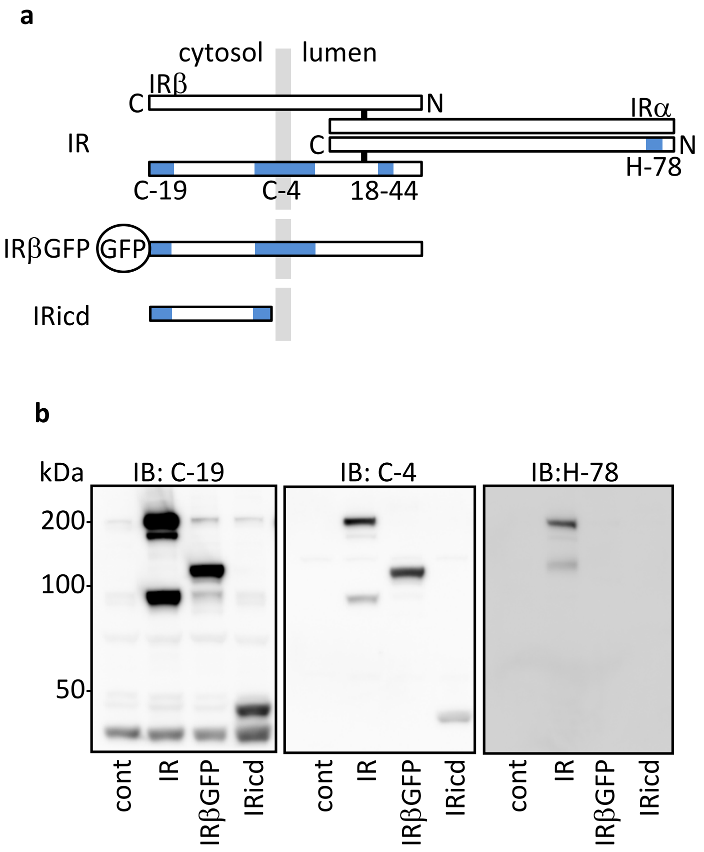

Supplementary Figure 1. Identification of IR regions recognized by various antibodies

Antibodies were tested for their ability to detect overexpressed wild type and truncated forms of IR. **a**. Schematic representation of the proteins used to test the antibodies: the full-length insulin receptor (IR), the GFP-tagged IR β-subunit (IRβGFP) and the intracellular part of IR (IRicd). The peptides used as antigens to produce the indicated antibodies (C-19; C-4; H-78) and the epitope recognized by the 18-44 antibody are positioned (blue rectangles). The α and β subunits are localized (IRα and IRβ, respectively) and their extremities (N, C) indicated. The membrane is represented by the light gray rectangles.

b. The proteins schematized in **a** were overexpressed in HEK 293 cells and detected by immunoblot (IB) using the indicated antibodies. The overexpressed proteins are indicated below the blot lanes (cont indicates that cells were transfected with an empty plasmid).

The C-19 antibody (raised against a peptide mapping at the C-terminus of IR β-subunit) allowed the detection of all overexpressed IR forms that contain the original C-terminus, *ie*: the full-length IR (the IR precursor that is not processed by furin was detected at around 200 kDa and IR β-subunit was detected at around 95 kDa); the GFP-tagged IR β-subunit (detected at around 120 kDa) and the IR intracellular domain (IRicd, detected at around 48 kDa). The monoclonal C-4 antibody (raised against amino acids 941-1010 of human IR β-subunit) detected all the IR forms, which means that it recognizes the N-terminal side of the intracellular domain of IR β-subunit. As expected, IR was detected by the H-78 antibody (raised against amino acids 128-205 in the IR α-subunit) only when the α-subunit was present (full-length IR).

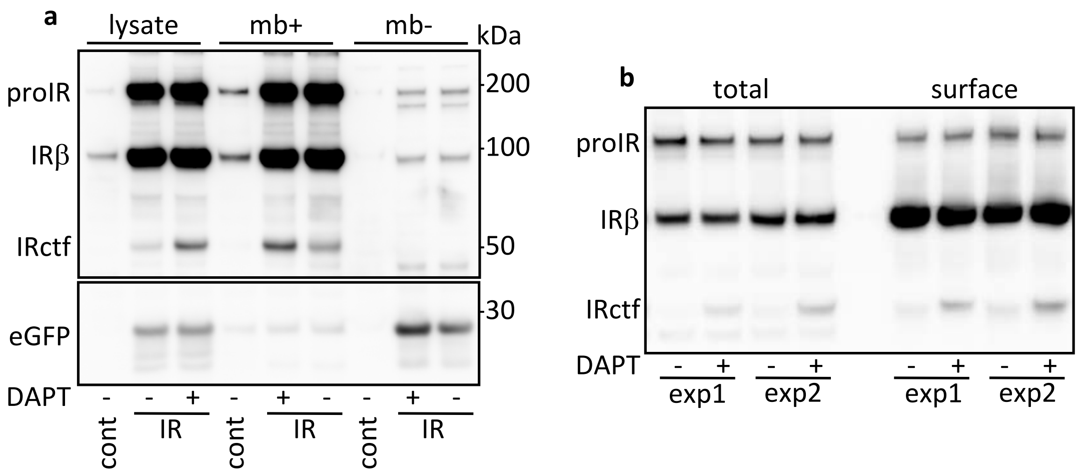

Supplementary Figure 2. IRctf is a transmembrane fragment of IR

a. HEK 293 cells were cotransfected with an empty plasmid (cont) or with the IR expression vector (IR) together with an eGFP expression vector then treated overnight (+) with DAPT. Cells were lysed in a hypotonic buffer and membranes from post-nuclear supernatant were pelleted by ultracentrifugation. IR and eGFP were detected by immunoblot in the total lysate, membrane-enriched (mb+) and membrane-depleted (mb-) fractions. The membrane-associated nature of the IRctf was demonstrated by its detection in membrane-enriched preparations (that also contained the pro and mature forms of IR), but not in the cytoplasmic extracts. As a control, the overexpressed cytoplasmic protein eGFP was mostly excluded from membrane-enriched preparations.

b. Cells were transfected with the IR expression vector and surface exposed proteins were labeled by biotinylation and purified. Total and cell surface proteins were submitted to immunoblot to detect IR using the C-19 antibody. Two independent experiments (exp1 and exp2) are shown. Fractions enriched in cell surface proteins (biotinylated proteins) contained proportionally more IR β-subunit and IRctf and less proIR than total cell lysates, which supports that IRctf, or a fraction of it, is at some point exposed at the cell surface and therefore IRctf is a transmembrane fragment of IR.

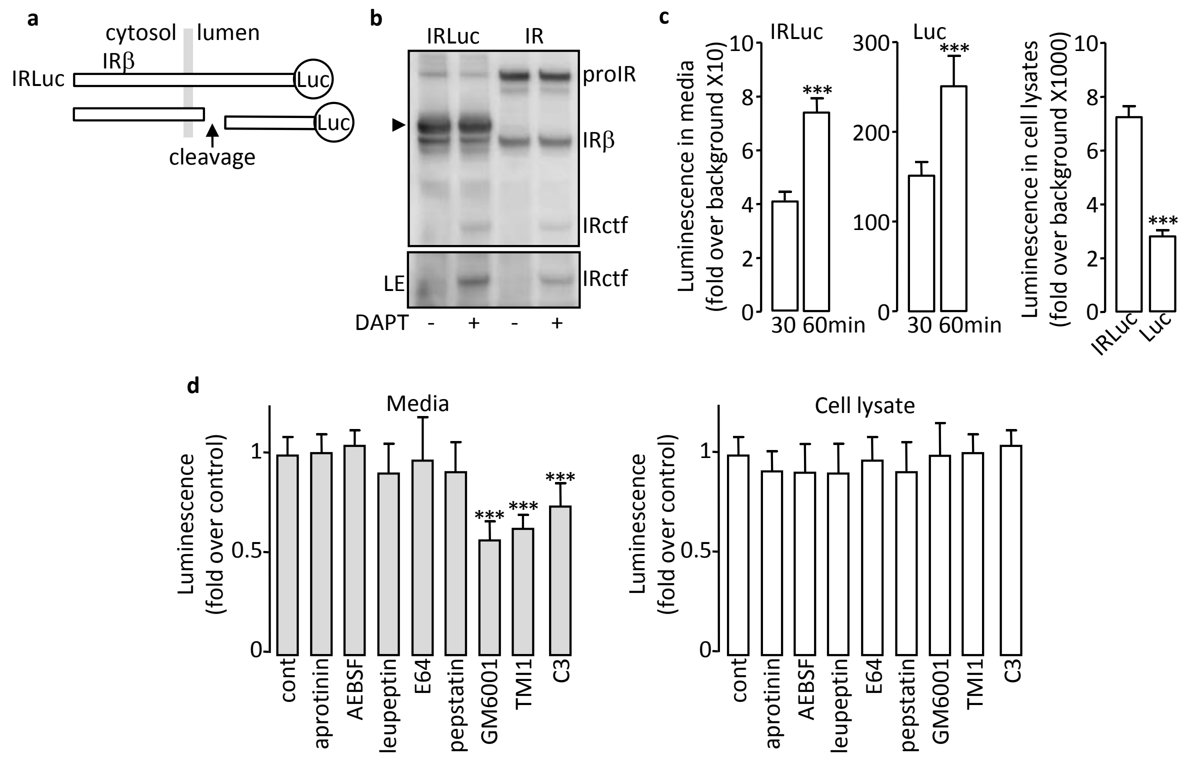

Supplementary Figure 3. Validation of the IR-cleavage reporter system

a. Schematic representation of the IR-cleavage reporter system (IRLuc), consisting of the Gaussia Luciferase (Luc) fused to the N-terminus of IR β-subunit.

b. IRctf was detected by immunoblot using the C-19 antibody in lysates of HEK 293 cells overexpressing IRLuc or wild type IR and treated with DAPT, indicating that IRLuc is efficiently cleaved. The arrowhead indicates the position of IRLuc. LE indicates long exposure of the immunoblot.

c. HEK 293 cells were transfected with vectors expressing IRLuc or Luc and luminescence was measured in the conditioned media after the indicated time of accumulation and in the cell lysates. The release of Luciferase in conditioned media indicates that IRLuc is efficiently cleaved and, as expected for a transmembrane protein, the accumulation of IRLuc in conditioned media was lower than that of the secreted Luciferase (Luc) while its amount in cell lysate was higher.

d. Cells expressing IRLuc were treated with the indicated protease inhibitors (10 μM, 16 h) then media were refreshed and luminescence was quantified in the media and in cell lysates after 3 hours. Luminescence is expressed as fold over the control (cont) situation. Broad spectrum inhibitors of serine (aprotinin and AEBSF), cysteine (leupeptin and E64) and aspartyl (pepstatin) proteases did not reduce IRLuc cleavage while inhibitors of metalloproteases (GM6001), ADAM17/MMP (TMI1) and the BACE1 inhibitor (C3) reduced the release of Luciferase.

Data are means ± s.d. Statistical analyses were made using unpaired t-test (c.) or ANOVA followed by Dunnett's test (d.). *** $p<0.001$.

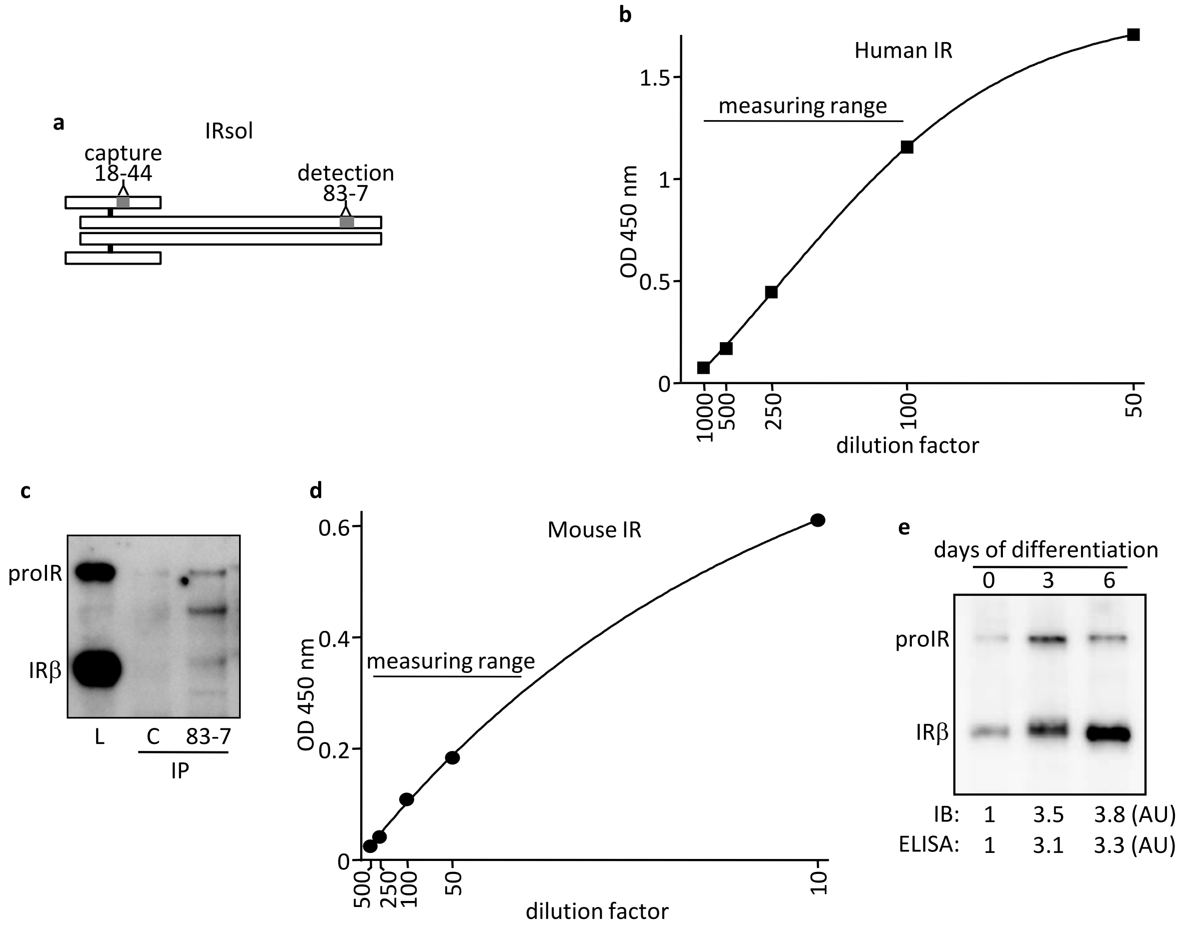

Supplementary Figure 4. Validation of the IR ELISA assay

a. Schematic representation of IRsol showing the position of the antibodies used for its detection by ELISA.

b. ELISA assay linearity for human IR was confirmed by testing serial dilutions (1:50 to 1:1000) of cell culture media from HEK 293 cells overexpressing human IR.

c. The ability of monoclonal 83-7 antibody to react with mouse IR was verified by performing immunoprecipitation (IP) of IR contained in the lysate (L) of 3T3-L1 mouse adipocytes followed by detection of the IRβ subunit by immunoblot. C: indicates no-antibody control IP.

d. ELISA assay linearity for mouse IR was confirmed by testing serial dilutions (1:10 to 1:500) of mouse 3T3-L1 adipocytes lysate .

e. Since the amount of protein in the ELISA test could bias the detection of mouse IR, we compared the ELISA and immunoblot measurements of IR contained in the same amount of total protein from lysates of undifferentiated 3T3-L1 mouse fibroblasts and after 3 and 8 days of adipocyte differentiation. As expected, ELISA and immunoblot show that the expression of IR increases during the adipocyte differentiation of 3T3L1 cells. Values from densitometric analysis of the immunoblot (IB) (proIR+IRβ) and values obtained with the ELISA are expressed relative to the undifferentiated situation and indicated below the blot.

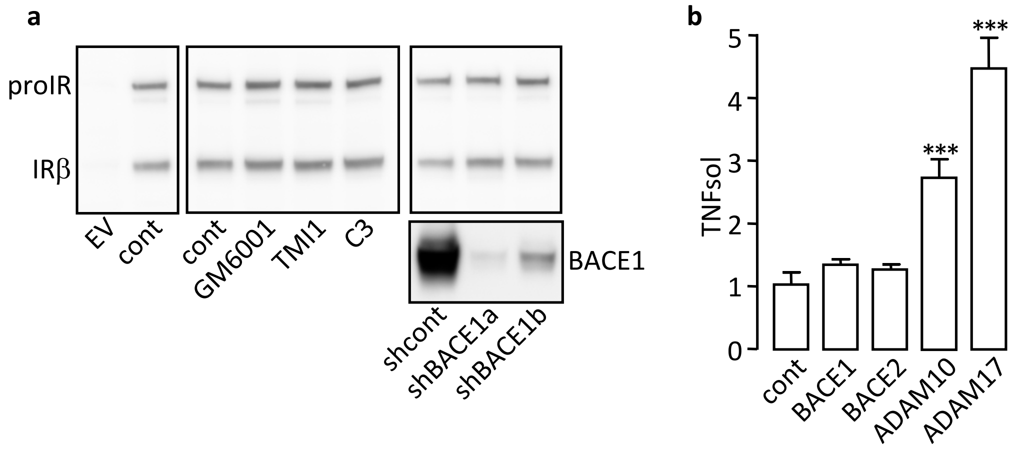

Supplementary Figure 5. BACE1 is involved in IR cleavage

a. Detection of IR by immunoblot in lysates of HEK 293 cells transfected with an empty vector (EV) or overexpressing IR and left untreated (cont) or treated with the indicated protease inhibitors (10 μM) or overexpressing IR together with different BACE1 specific shRNA (shBACE1a, shBACE1b) or a control shRNA (shcont), shows that these treatments do not alter IR expression. The knockdown efficiency of the two BACE1 shRNA was validated by the detection of BACE1 in the lysate of cells coexpressing BACE1 and its specific shRNA.

b. The activity of overexpressed ADAM10 and ADAM17 was confirmed by their ability to increase the cleavage of TNFα, which was measured by ELISA in conditioned media of cells transfected with the TNFα expression vector together with an empty plasmid (cont), BACE1, BACE2, ADAM10 or ADAM17. Data are means ± s.d. Statistical analysis was made using ANOVA followed by Dunnett's test. *** $p<0.001$.

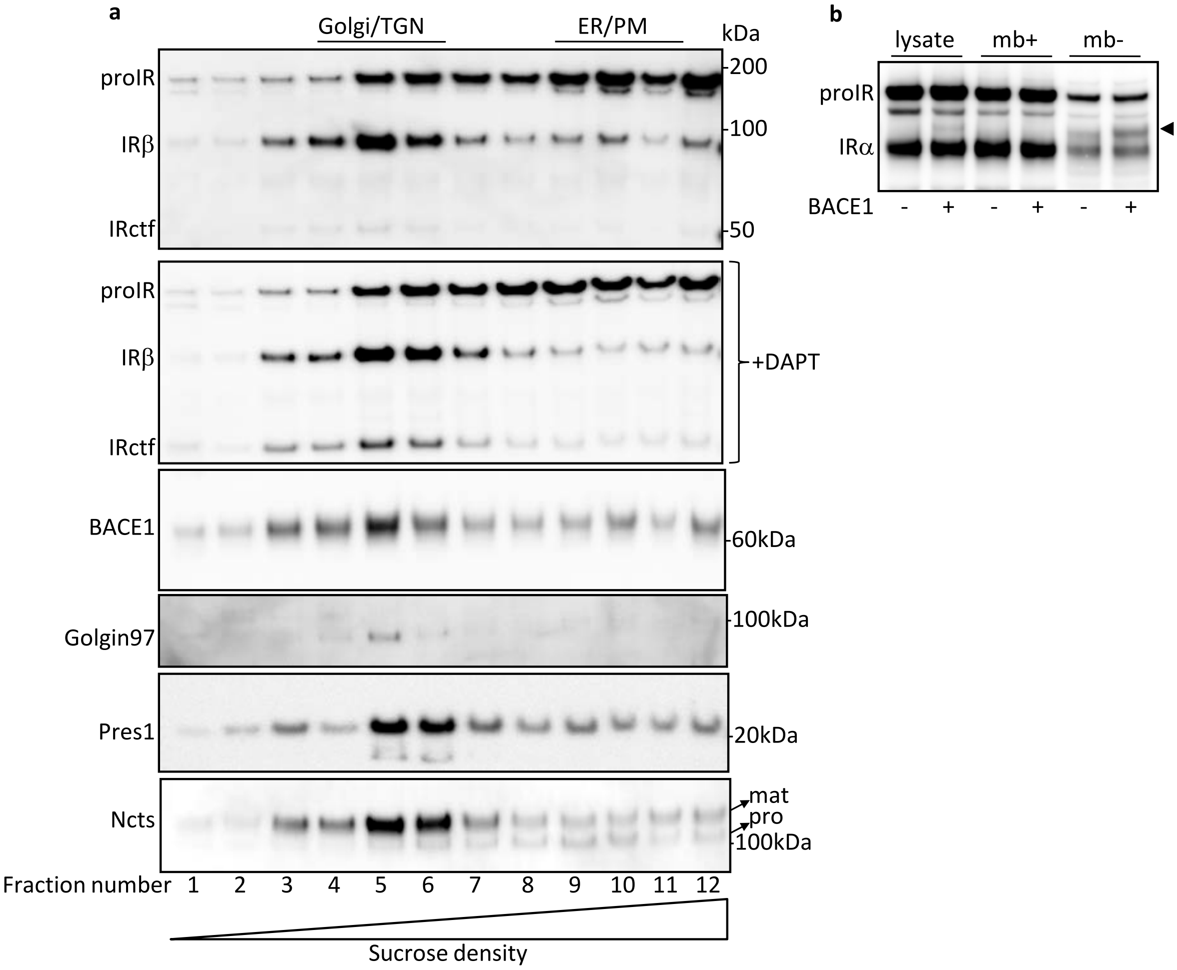

Supplementary Figure 6. BACE1 and IR co-purify in the Golgi/TGN

a. HEK 293 cells expressing BACE1 and IR were treated or not with DAPT then homogenized and post-nuclear supernatants were fractionated on an equilibrium sucrose gradient, as previously done to demonstrate BACE1 residence in the Golgi/TGN [1]. IR, BACE1, Golgin 97, Presenilin 1 (Pres1) and Nicastrin (Ncts) were detected by immunoblot in each fraction. Proform (pro) and mature form (mat) of Nicastrin are indicated. BACE1 is present throughout the gradient with high enrichment in the Golgi/TGN fractions (identified by Golgin 97 detection). As previously shown [2], Presenilin 1 and mature Nicastrin (two proteins of the γ-secretase complex) are mainly detected in Golgi/TGN-enriched fractions, while mature and immature Nicastrin are detected in endoplasmic reticulum/plasma membrane-enriched fractions (ER/PM). Most of the mature IR is found in Golgi/TGN-enriched fractions, consistent with IR proteolytic maturation by furin in the TGN [3-6].

b. Cells expressing IR and BACE1 (+) or inactive BACE1 (-) were homogenized, membranes were pelleted by ultracentrifugation of post-nuclear supernatants and IR α-subunit was detected by immunoblot in total cell lysates, membrane-enriched (mb+) and membrane-depleted (mb-) fractions. A protein migrating slower than the IR α-subunit is detected in total cell lysates and membrane-depleted fractions (arrowhead) but not in membrane-enriched fractions from cells overexpressing the active form of BACE1. This protein is detected by the antibody specific for IR α-subunit, migrates slower than the cellular IR α-subunit, is not membrane attached and its amount increases upon active BACE1 overexpression. All these features are those of IRsol, confirming that IRsol is generated inside the cell.

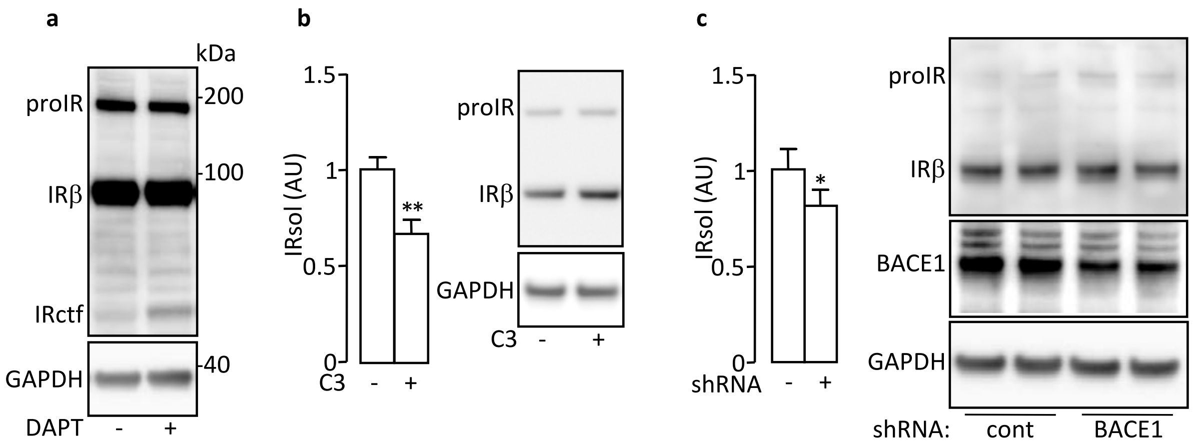

Supplementary Figure 7. BACE1-dependent cleavage of endogenous IR

a. Human hepatoma HepG2 cells were treated overnight with DAPT and the expression of endogenous IR and GAPDH (loading control) were analyzed by immunoblot. HepG2 cells express substantial amount of endogenous IR and cleave the ectodomain of IR as evidenced by the generation of IRctf.

b. HepG2 cells were treated overnight with the BACE1 inhibitor (C3) then IRsol in conditioned media was measured by ELISA (left) and the cellular expression of IR was analyzed by immunoblot (right). Treatment with the BACE1 inhibitor reduced the amount of IRsol and slightly increased the expression levels of the mature form of IR.

c. HepG2 cells were transfected with control (-) or BACE1-specific shRNA (+) then IRsol in conditioned media was measured by ELISA (left) and IR, BACE1 and GAPDH were analyzed by immunoblot (right). The low transfection efficiency of HepG2 is probably responsible for the partial knockdown of BACE1, as a consequence IRsol is poorly reduced.

Data are means ± s.d. Statistical analysis was made using unpaired t-test. * $p<0.05$, ** $p<0.01$.

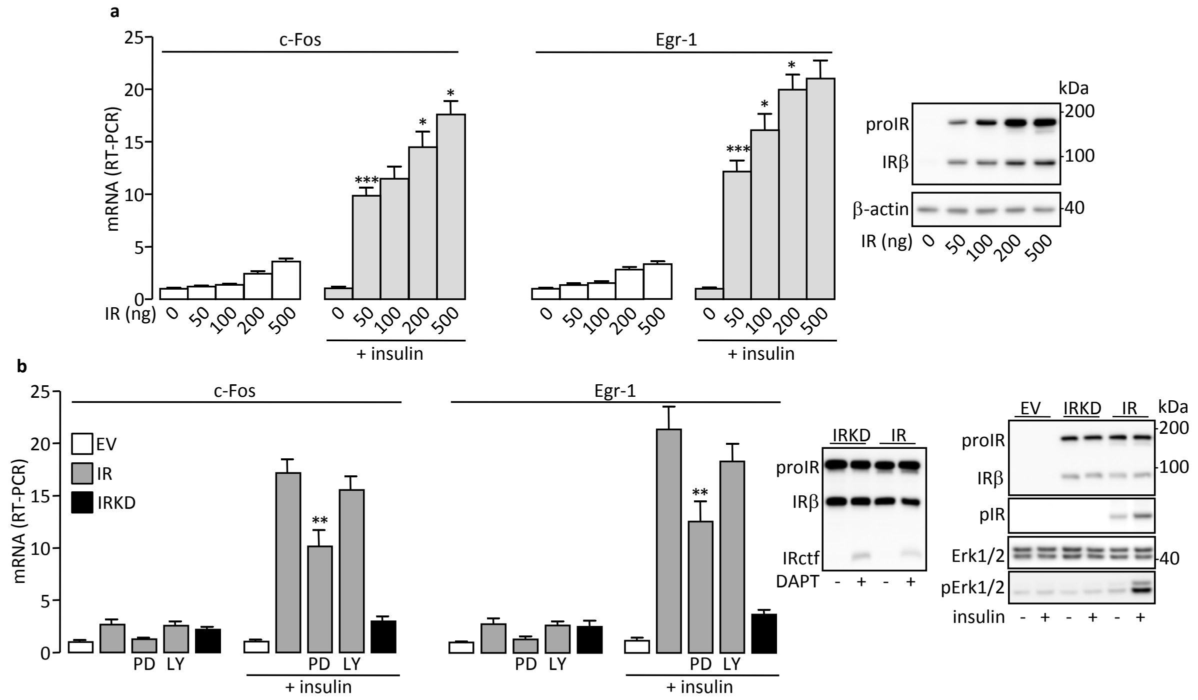

Supplementary Figure 8. Expression of insulin-dependent immediate-early genes

a. HEK 293 cells were transfected with increasing amount of IR coding vector or **b.** with an empty vector (EV), a vector coding for the wild type IR (IR) or for a kinase dead mutated form of IR (Y_3F; IRKD). Then cells were serum deprived for 20 hours and stimulated with insulin (5 nM , 45 minutes). Where indicated cells were treated for 30 minutes prior insulin stimulation with 10 μM of the Erk1/2 pathway inhibitor PD98059 (PD) or the PKB pathway inhibitor LY294002 (LY). Levels of c-Fos and EGR-1 mRNA were measured by RT-PCR. Inset in **a.** shows the amount of overexpressed IR following transfection. Insets in **b.** show that IRKD is efficiently overexpressed and cleaved (as judged by IRctf generation) but is not phosphorylated following insulin stimulation. Furthermore, overexpression of IR increased basal and insulin-stimulated phosphorylation of IR and Erk1/2. Data are means ± s.d. expressed as fold over the situation without IR overexpression nor insulin stimulation. Statistical analyses were made using ANOVA followed by Bonferonni's t-test. * $p<0.05$, ** $p<0.01$, *** $p<0.001$ (in a. : vs the immediately lower insulin concentration; in b. : vs IR in the presence of insulin)

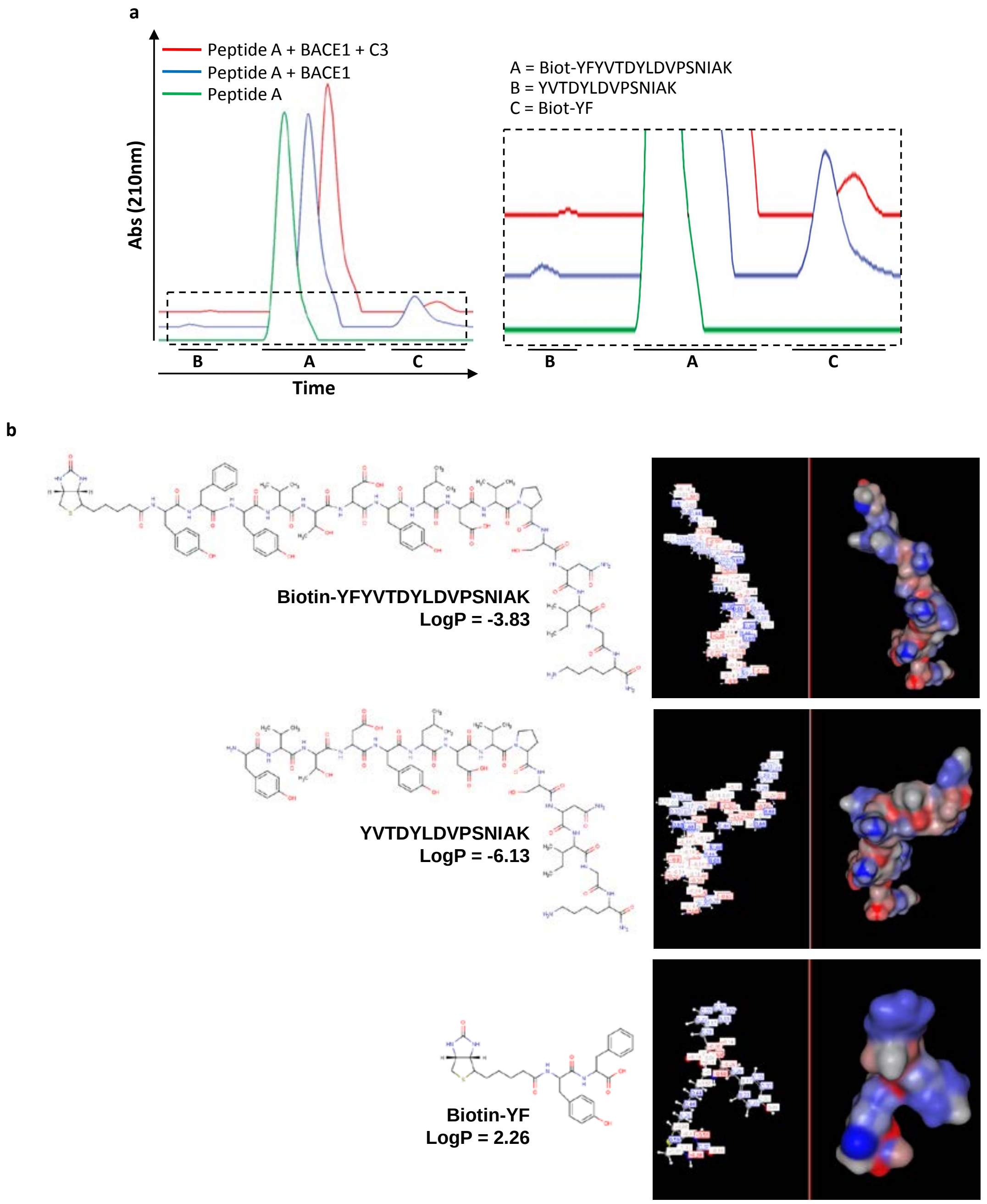

Supplementary Figure 9. *In vitro* cleavage of IR-mimetic peptide by recombinant BACE1

a. The N-biotinilated synthetic peptide corresponding to the extracellular stalk of IR and containing part of the BACE1 target motif (peptide A) was incubated without (green curve) or with recombinant active soluble BACE1 (from Enzo Life Sciences in the conditions described by the manufacturer to measure BACE1 specific activity), in the absence (blue curve) or presence of BACE1 inhibitor C3 (red curve), then the reaction products were separated by RP-HPLC (Agilent 1260 infinity; C18 column; mobile phases: water + 0.1% formic acid / acetonitrile + 0.1% formic acid. The presence of BACE1 allows the generation of fragments (B and C), which is diminished in the presence of BACE1 inhibitor. The framed area is magnified on the right panel.

b. Evaluation of the LogP values (Marvin SKETCH 6.1.3) of the original peptide and fragments potentially generated by BACE1 activity suggests that, in our RP-HPLC settings, the fragments of the original peptide would be eluted before and after the original peptide, which is in agreement with the RP-HPLC profiles obtained.

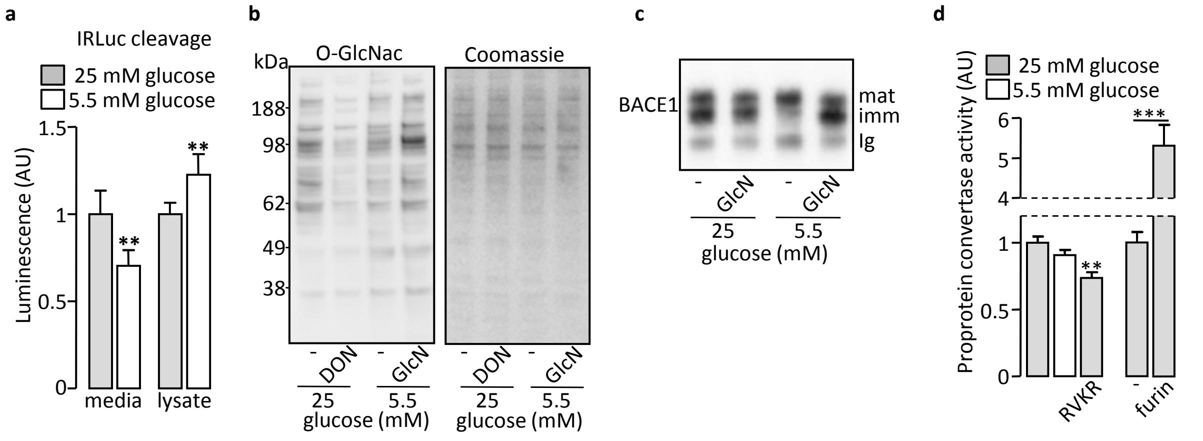

Supplementary Figure 10. Effect of glucose on IR cleavage and proprotein convertase activity

a. HEK 293 cells expressing the IR cleavage reporter system (IRLuc) and BACE1 were incubated for 17 h in media containing the indicated glucose concentrations then media were refreshed and luminescence was quantified in the media and in cell lysates after 5 hours.

b. Cells were incubated for 24 h in media containing the indicated glucose concentrations in the absence (-) or presence of deoxynorleucine (DON; 5mM) or Glucosamine (GlcN; 1mM) and cell lysates were analyzed by SDS-PAGE followed by immunoblot for the detection of global cellular O-GlcNacylation (O-GlcNac) or by staining with Coomassie blue (for total protein visualisation).

c. Cells were incubated for 24 h in media containing the indicated glucose concentrations in the absence (-) or presence of Glucosamine (GlcN; 1mM) and BACE1 was analyzed by immunoblot after immunoprecipitation. Positions of the heavy chain of the precipitating antibody (Ig), immature (imm) and mature (mat) BACE1 are indicated.

d. Proprotein convertase activity was measured after transfection of cells with the intra Golgi proprotein convertase activity reporter system [7]. Cells were incubated for 17 h in media containing the indicated glucose concentrations, media were refreshed then secreted embryonic alkaline phosphatase (SEAP) activity was quantified in the media and in cell lysates after 5 hours. Data are ratios of secreted / cellular SEAP. As controls for the specific measurement of convertase activity, treatment with proprotein convertase inhibitor (dec-RVKR-cmk: RVKR; 50 μM for 24 h) reduces the release of SEAP, while overexpression of furin increases the release of SEAP compared to cells transfected with empty vector (-).

Statistical analyses were made using unpaired t-test. **p<0.01, *** p<0.001.

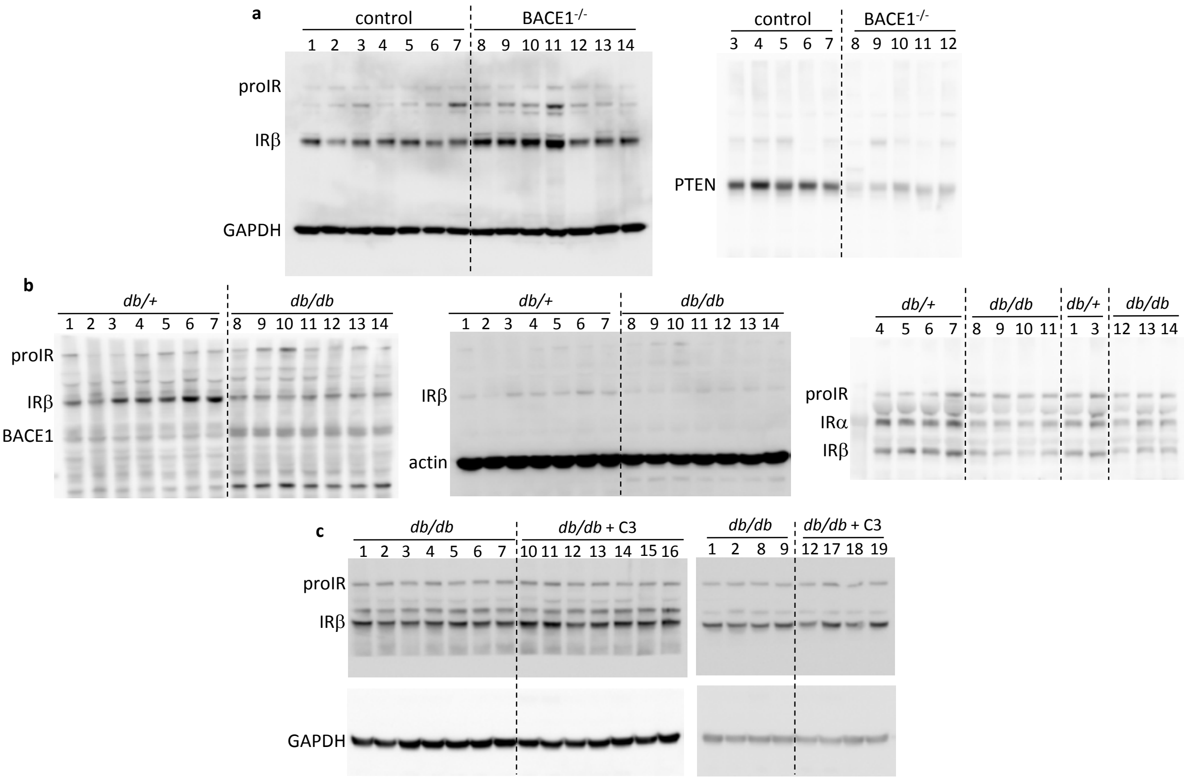

Supplementary Figure 11. Representative uncropped images of immunoblots used for the densitometric analyses.

a. IR, PTEN and GAPDH levels in the liver of control and BACE1$^{-/-}$ mice. **b.** IR, BACE1 and actin levels in the liver of *db/+* and *db/db* mice. **c.** IR and GAPDH levels in the liver of *db/db* mice untreated or treated with C3 (+C3). Immunoblots with an exposure time allowing to visualize the position of the proteins of interest on the same membrane as well as the background inherent to their detections are shown. Samples are identified by the number indicated above the blot.

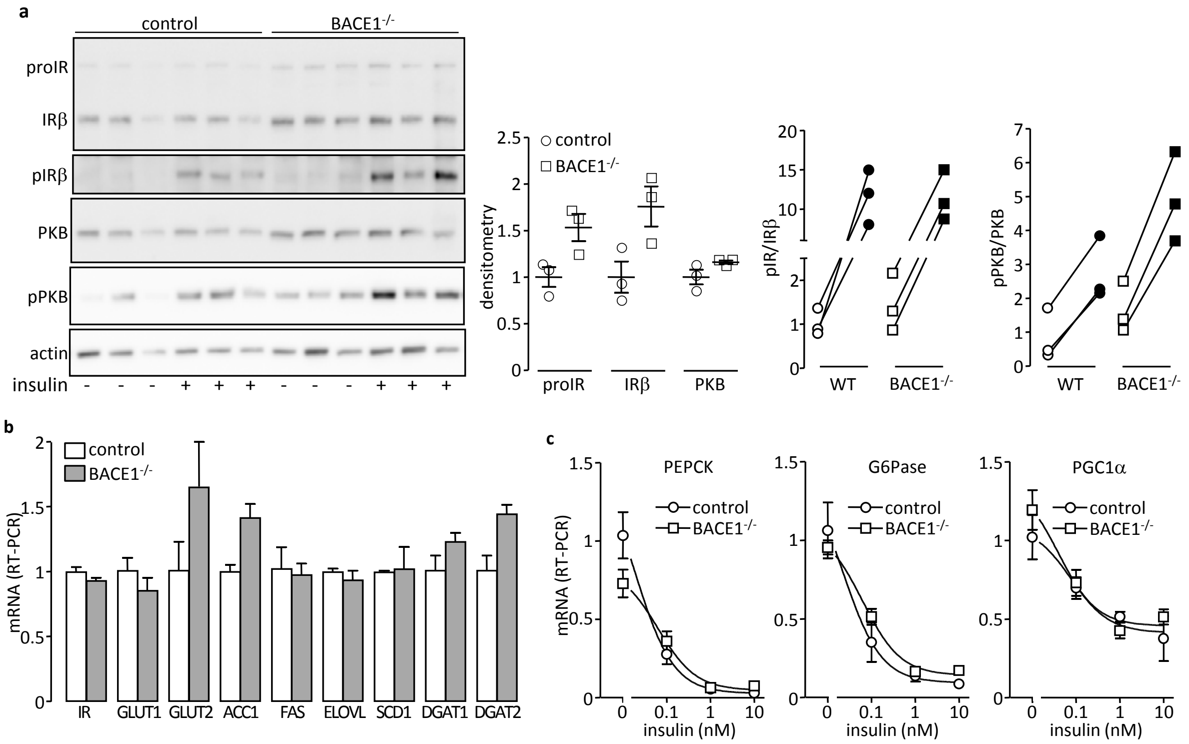

Supplementary Figure 12. Expression of several proteins and genes in BACE1$^{-/-}$ hepatocytes

a. Primary hepatocytes isolated from BACE1$^{-/-}$ mice (n = 3) and from their control littermates (n = 3) were stimulated with insulin (+; 10 nM, 10 minutes) and IR, PKB, their phosphorylated forms (pIRβ and pPKB) and β-actin (loading control) were analyzed by immunoblot. A densitometric analysis of the indicated protein bands normalized to β-actin and of IR and PKB phosphorylation was performed (right). The values obtained from the hepatocytes originating from the same mouse are connected by a line.

b. The levels of the indicated mRNA were quantified by RT-PCR in control (n = 4) and BACE1$^{-/-}$ (n = 3) primary hepatocytes. Data are means ± s.d. expressed relative to control hepatocytes (no difference is significant).

c. mRNA levels of PEPCK, G6PASE and PGC1α were measured in control and BACE1$^{-/-}$ primary hepatocytes stimulated for 6 hours with the indicated concentrations of insulin (curves from control and BACE1$^{-/-}$ hepatocytes are not different , F test).

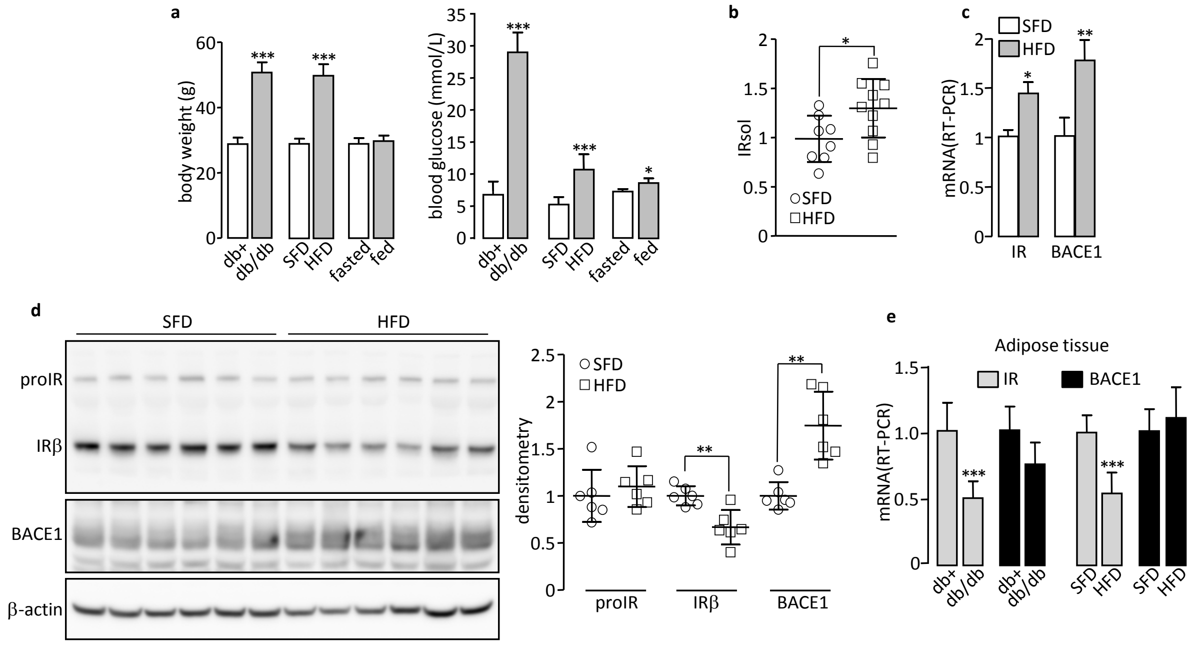

Supplementary Figure 13. Biological parameters of the mouse models and IR cleavage in glucose intolerant mice

a. Body weight and blood glucose of the mouse models used in this study (standard-fat and high-fat diet fed mice: SFD and HFD, respectively). Plasma levels of IRsol (**b**), liver mRNA levels (**c**) and protein expression (**d**) of IR and BACE1 were measured in SFD (n = 10) and HFD (n = 10) mice. A densitometric analysis relative to β-actin (loading control) of the proIR, IRα, IRβ and BACE1 bands was performed (d, right), values are expressed relative to the means of the wild type mice. **e**. mRNA levels of IR and BACE1 were measured by RT-PCR in the epididymal fat of db+, db/db, SFD and HFD fed mice. Data are means ± s.d. Statistical analyses were made using unpaired t-test. * $p<0.05$, ** $p<0.01$, *** $p<0.001$.

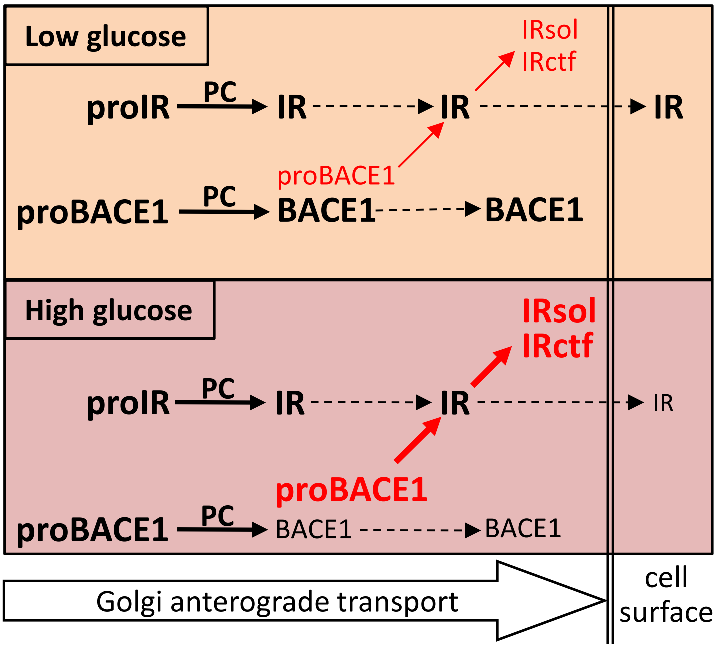

Supplementary Figure 14: Model for the regulation of BACE1-dependent IR cleavage by glucose
In the secretory pathway, proprotein convertases (PC) cleave the propeptides of immature forms of IR and BACE1 (proIR, proBACE1) giving their mature forms. However, a certain amount of immature BACE1 escapes the PC-dependent maturation. This immature BACE1 is responsible for IR cleavage. In the presence of low glucose concentration, only a small amount of BACE1 is not matured by PC, thus IR is poorly cleaved and an optimal amount of IR reaches the cell surface. High glucose increases the amount of BACE1 that escapes PC-dependent maturation, which increases IR cleavage and reduces the amount of IR that reaches the cell surface.

Supplementary Table 1:

Wild type and mutated sequences of the region of IR β-subunit cleaved by BACE1

<table>
<tr><td>Subsites</td><td></td><td></td><td></td><td>P_7</td><td>P_6</td><td>P_5</td><td>P_4</td><td>P_3</td><td>P_2</td><td>$\mathbf{P_1}$</td><td>$\mathbf{P'_1}$</td><td>P'_2</td><td>P'_3</td><td>P'_4</td><td></td><td></td><td></td><td></td><td></td><td></td><td></td><td></td><td></td><td></td><td></td></tr>
<tr><td>wild type</td><td>N</td><td>G</td><td>S</td><td>W</td><td>T</td><td>E</td><td>P</td><td>T</td><td>Y</td><td>F</td><td>Y</td><td>V</td><td>T</td><td>D</td><td>Y</td><td>L</td><td>D</td><td>V</td><td>P</td><td>S</td><td>N</td><td>I</td><td>A</td><td>K</td><td>||</td></tr>
<tr><td>$W_{936}H$</td><td>N</td><td>G</td><td>S</td><td>H</td><td>T</td><td>E</td><td>P</td><td>T</td><td>Y</td><td>F</td><td>Y</td><td>V</td><td>T</td><td>D</td><td>Y</td><td>L</td><td>D</td><td>V</td><td>P</td><td>S</td><td>N</td><td>I</td><td>A</td><td>K</td><td>||</td></tr>
<tr><td>$T_{937}M$</td><td>N</td><td>G</td><td>S</td><td>W</td><td>M</td><td>E</td><td>P</td><td>T</td><td>Y</td><td>F</td><td>Y</td><td>V</td><td>T</td><td>D</td><td>Y</td><td>L</td><td>D</td><td>V</td><td>P</td><td>S</td><td>N</td><td>I</td><td>A</td><td>K</td><td>||</td></tr>
<tr><td>$F_{942}K/Y_{943}K$</td><td>N</td><td>G</td><td>S</td><td>W</td><td>T</td><td>E</td><td>P</td><td>T</td><td>Y</td><td>K</td><td>K</td><td>V</td><td>T</td><td>D</td><td>Y</td><td>L</td><td>D</td><td>V</td><td>P</td><td>S</td><td>N</td><td>I</td><td>A</td><td>K</td><td>||</td></tr>
<tr><td>Positions</td><td>933</td><td></td><td></td><td></td><td></td><td></td><td></td><td></td><td></td><td></td><td></td><td></td><td></td><td></td><td></td><td></td><td></td><td></td><td></td><td></td><td></td><td></td><td></td><td>956</td><td></td></tr>
</table>

The sequence of the extracellular region of IR β-subunit identified in this work as being cleaved by BACE1 is located between amino acids 933 and 956 and that identified by bioinformatics [8] is located between amino acids 939 and 946. Wild type and mutated sequences are shown. Substituted amino acids are underlined. Amino acids believed to be involved in the recognition by BACE1 are highlighted in grey and the predicted P_7 to P'_4 subsites are indicated, amino acids of the potential scissible bound are in bold. **||** indicates the membrane.

Supplementary Methods

3T3-L1 cell culture and adipocyte differentiation

3T3-L1 cells (from ATCC) were routinely cultured in DMEM with 4 mM l-glutamine, 4.5 g/L glucose, 0.11 g/L sodium pyruvate, and supplemented with 10% fetal bovine serum plus antibiotics. Two days after confluence, preadipocytes were staged to differentiate by changing the medium to one containing the induction mixture (0.1 μM dexamethazone , 500 μM 3-Isobutyl-1-methylxanthine, and 174.5 nM insulin). After 48 h (day 0 of differentiation), the medium was removed and replaced by DMEM containing 174.5 nM insulin.

Cell surface protein biotinylation and isolation

The Cell Surface Protein Isolation Kit was used as described by the manufacturer (Pierce, Rockford, IL, USA). In brief, cells were washed with ice-cold PBS. The membrane-impermeable biotinylation reagent, EZ-Link Sulfo-NHS-SS-Biotin was added to a final concentration of 100 µM and the cells were incubated on ice for 30 min. The biotinylation reaction was stopped by adding the quenching solution followed by two PBS washes. The cells were lysed and labeled proteins were isolated with NeutrAvidin Agarose resin. Proteins were eluted from the resin and analyzed by immunoblot.

IR-cleavage reporter system analysis

HEK 293 cells were transfected with the expression vector for IR β-subunit N-terminally fused to Gaussia Luciferase. Luciferase activity in conditioned media and cell lysates was measured after adding the Genofax C reagent from Yelen (Ensues la Redonne, France) with the Victor 4 luminometer (Perkin Elmer).

Sucrose Density Gradient Fractionation

The separation of TransGolgi Network and the Endoplasmic Reticulum membranes was performed as previously described [1]. Briefly, cells, in homogenizing buffer (10 mM Tris pH 7.4, 1mM Mg(Ac)2, and protease inhibitor mixture), were passed through a 28 gauge needle (10 strokes), a post nuclear supernatant was prepared and adjusted to 0.25 M sucrose (final volume 1 mL). The homogenate was loaded onto the top of a step gradient comprised of 1.5 mL of 2 M sucrose, 2.5 mL of 1.3 M sucrose, 2.5 mL of 1.16 M sucrose, 2 mL of 0.8 M sucrose, 2 mL of 0.5 M sucrose, and 0.5 mL of 0.25 M sucrose. The gradients were centrifuged for 2.5 h at 100,000 × *g* in a Beckman SW41Ti rotor. Twelve 1-mL fractions were collected from the top of the gradient. Equal volumes of solution from each fraction were separated on SDS-PAGE.

Synthesis of TMI1

General synthetic pathway for TMI1

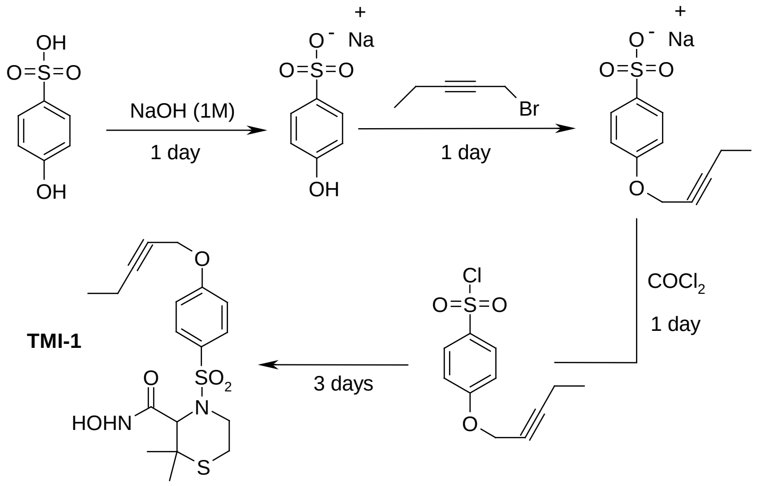

Preparation of 4-hydroxy benzenesulfonate sodium salt

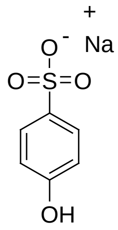

1 equivalent (13.4 mL) of 4-hydroxybenzene sulfonic acid (50% water) and 1 equivalent of 1 M NaOH (50 mL) were mixes in round flask. After stirring for 24 hours, the crude residue was poured in acetone. After filtration of the white solid, this latter is dried in vacuo for 3 hours.

Preparation of 4-But-2-ynyloxy- benzenesulfonic acid sodium salt

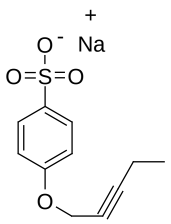

To a solution of 5.2 g (0.022 mol) of 4-hydroxybenzenesulfonate sodium salt in 100 mL of isopropanol and 23 mL of a 1N solution of sodium hydroxide was added 6 g (0.45 mol) of 1-bromo-2-butyne. The resulting mixture was heated to 70°C for 24h and then isopropanol removed by evaporation under vacuum. The resulting white precipitate was collected by filtration, washed with isopropanol and diethylether and dried in vacuo to afford 5.3 g (93%) of the expected butynylether as a white solid.

Preparation of 4-But-2-ynyloxy- benzenesulfonyl chloride

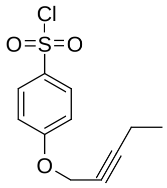

To a solution of 4.3 mL (0.009 mol) of 2M oxalylchloride/dichloromethane solution at 0°C in 10 mL of dichloromethane was added dropwise 677 µL (0.0087 mol) of DMF followed by 724 mg (0.0029 mol) of 4-but-2-ynyloxy-benzenesulfonic sodium salt. The reaction was stirred for 1hour a 0°C then let warm to room temperature for 2 days. The reaction was poured in to ice and extracted with petroleum ether (100 mL). The organic phases were washed with water and brine, dried over Na_2SO_4, filtered, concentrated under vacuum to afford the expected compound as a yellow powder in 84% yield.

Preparation of TMI1

TMI-1

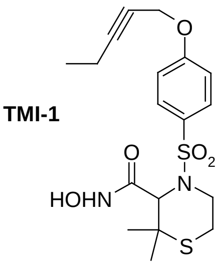

A suspension of (3S)-2,2-dimethyl-tetrahydro-2H-1,4-thiazine-3- arboxylic acid (1.7g, 0.01 mol) in dichloromethane (25 mL) in a 200 mL 3 necked round flask equipped with a condenser and a magnetic stirringwas heated to reflux for 15 minutes. BSTFA (5.15 g, 0.02 mol) was added over 30 minutes and the mixture stirred at reflux until a clear solution was obtained. This solution was cooled to 0°C then N methylmorpholine (1.43 mL, 0.013 mol) was added over 10 minutes. A solution of Sulfonyl chloride (3g, 0.01 mol) in dichloromethane (30 mL) was added maintaining the temperature at 5°C. After 1h of stirring the ice bath was removed and the solution was allowed to warm to room temperature. After overnight stirring, the solution was cooled to 5°C and DMF (300µL, 0.04mol) was added with a syringe followed by oxalyl chloride (3.1 g, 0.02 mol). The reaction mixture was warmed to room temperature and stirred for 24h. THF (15 mL), water (10 mL) and 50% aq. solution of hydroxylamine (4.3 mL, 0.07 mol) were added maintaining the temperature at 5°C. The solution was warmed to room temperature and stirring was maintained for 10 hours. Water was added and ethylacetate used to perform an extraction. The organic phase was dried over Na2SO4, filtered and concentrated under vacuum before purification the crude residue by column chromatography

(eluent petroleum ether / ethylacetate) affording the expected TMI1 in 60% yield as a light beige solid.

Supplementary References

1. Yan, R., Han, P., Miao, H., Greengard, P. & Xu, H. The transmembrane domain of the Alzheimer's beta-secretase (BACE1) determines its late Golgi localization and access to beta - amyloid precursor protein (APP) substrate. *J Biol Chem* **276**, 36788-36796 (2001).
2. Leem, J.Y. *et al.* Presenilin 1 is required for maturation and cell surface accumulation of nicastrin. *J Biol Chem* **277**, 19236-19240 (2002).
3. Bass, J., Turck, C., Rouard, M. & Steiner, D.F. Furin-mediated processing in the early secretory pathway: sequential cleavage and degradation of misfolded insulin receptors. *Proc Natl Acad Sci U S A* **97**, 11905-11909 (2000).
4. Robertson, B.J., Moehring, J.M. & Moehring, T.J. Defective processing of the insulin receptor in an endoprotease-deficient Chinese hamster cell strain is corrected by expression of mouse furin. *J Biol Chem* **268**, 24274-24277 (1993).
5. Yoshimasa, Y., Paul, J.I., Whittaker, J. & Steiner, D.F. Effects of amino acid replacements within the tetrabasic cleavage site on the processing of the human insulin receptor precursor expressed in Chinese hamster ovary cells. *J Biol Chem* **265**, 17230-17237 (1990).
6. Hedo, J.A., Kahn, C.R., Hayashi, M., Yamada, K.M. & Kasuga, M. Biosynthesis and glycosylation of the insulin receptor. Evidence for a single polypeptide precursor of the two major subunits. *J Biol Chem* **258**, 10020-10026 (1983).
7. Bernot, D. *et al.* Plasminogen activator inhibitor 1 is an intracellular inhibitor of furin proprotein convertase. *J Cell Sci* **124**, 1224-1230 (2011).
8. Johnson, J.L., Chambers, E. & Jayasundera, K. Application of a Bioinformatics-Based Approach to Identify Novel Putative in vivo BACE1 Substrates. *Biomed Eng Comput Biol* **5**, 1-15 (2013).
